# EfficientNet-B3-Based Automated Deep Learning Framework for Multiclass Endoscopic Bladder Tissue Classification

**DOI:** 10.3390/diagnostics15192515

**Published:** 2025-10-03

**Authors:** A. A. Abd El-Aziz, Mahmood A. Mahmood, Sameh Abd El-Ghany

**Affiliations:** Department of Information Systems, College of Computer and Information Sciences, Jouf University, Sakaka 72388, Saudi Arabia; mamahmood@ju.edu.sa (M.A.M.); saabdelwahab@ju.edu.sa (S.A.E.-G.)

**Keywords:** bladder cancer, deep learning, endoscopic images, EfficientNet-B3, Endoscopic Bladder Tissue Classification dataset

## Abstract

**Background:** Bladder cancer (BLCA) is a malignant growth that originates from the urothelial lining of the urinary bladder. Diagnosing BLCA is complex due to the variety of tumor features and its heterogeneous nature, which leads to significant morbidity and mortality. Understanding tumor histopathology is crucial for developing tailored therapies and improving patient outcomes. **Objectives:** Early diagnosis and treatment are essential to lower the mortality rate associated with bladder cancer. Manual classification of muscular tissues by pathologists is labor-intensive and relies heavily on experience, which can result in interobserver variability due to the similarities in cancerous cell morphology. Traditional methods for analyzing endoscopic images are often time-consuming and resource-intensive, making it difficult to efficiently identify tissue types. Therefore, there is a strong demand for a fully automated and reliable system for classifying smooth muscle images. **Methods:** This paper proposes a deep learning (DL) technique utilizing the EfficientNet-B3 model and a five-fold cross-validation method to assist in the early detection of BLCA. This model enables timely intervention and improved patient outcomes while streamlining the diagnostic process, ultimately reducing both time and costs for patients. We conducted experiments using the Endoscopic Bladder Tissue Classification (EBTC) dataset for multiclass classification tasks. The dataset was preprocessed using resizing and normalization methods to ensure consistent input. In-depth experiments were carried out utilizing the EBTC dataset, along with ablation studies to evaluate the best hyperparameters. A thorough statistical analysis and comparisons with five leading DL models—ConvNeXtBase, DenseNet-169, MobileNet, ResNet-101, and VGG-16—showed that the proposed model outperformed the others. **Conclusions:** The EfficientNet-B3 model achieved impressive results: accuracy of 99.03%, specificity of 99.30%, precision of 97.95%, recall of 96.85%, and an F1-score of 97.36%. These findings indicate that the EfficientNet-B3 model demonstrates significant potential in accurately and efficiently diagnosing BLCA. Its high performance and ability to reduce diagnostic time and cost make it a valuable tool for clinicians in the field of oncology and urology.

## 1. Introduction

Cancer remains one of the primary causes of death worldwide, accounting for 9.6 million fatalities in 2017 [1]. BLCA, also referred to as urological cancer, affects the urinary bladder [2]. The bladder is a hollow organ located in the lower abdomen, primarily responsible for storing urine that is collected from the kidneys through the ureters until it is expelled from the body [2]. When not filled, it sits behind the pubic symphysis, and when filled, it ascends into the abdominal cavity [3]. It has four distinct surfaces: one superior, one posterior (the bladder floor), and two inferolateral surfaces [4].

BLCA, recognized as a significant type of urological cancer, resulted in 196,500 fatalities and ranked as the 9th leading cause of cancer-related deaths in males and the 19th in females [5,6]. In 2018, there were 549,393 new diagnoses globally, with 199,922 individuals losing their lives to the disease. While the age-standardized incidence rate (ASIR) varies greatly across different regions, it is anticipated to continue increasing over the next ten years [7]. BLCA represents 3% of cancer diagnoses globally and is particularly common in developed countries. In the United States (US), it ranks as the sixth most frequently diagnosed cancer. Approximately 90% of BLCA cases occur in individuals aged 55 and older, with men being four times more likely to be affected than women. Although the average survival rate over five years in the US is 77%, this rate drops significantly to just 5% for patients with metastatic disease [5]. It is not surprising that 90% of BLCA cases, particularly in developed countries, originate from urothelial cells, primarily found in the bladder, although they can occasionally occur in the urinary tract. Localized urothelial cancer has a very favorable prognosis; however, if it invades the smooth muscle, the survival rates decrease significantly. The remaining 10% of BLCA cases are squamous cell types, which are more common in Africa and are likely linked to the protozoan infection schistosomiasis [6].

BLCA is defined by two main factors: its grade, which indicates how abnormal cancer cells appear, and its stage, which reflects the extent of tumor invasion through the bladder wall. By grade, it is categorized into: Low-grade and High-grade [8].

By stage, BLCA is divided into non-muscle-invasive bladder cancers (NMIBCs), which remain confined to the mucosa or submucosa, and muscle-invasive bladder cancers (MIBCs), which penetrate into or through the detrusor muscle [8]. Patients with NMIBC have a good prognosis with a 5-year BLCA-specific mortality of 0.5%, 1.7% and 6.8% among patients with grade 1, 2, and 3 tumors, respectively [9].

Symptoms of BLCA [10], such as blood in the urine, include changes in urination (Irritative bladder symptoms), and pelvic or lower back pain. If the cancer grows larger or spreads (metastasizes), symptoms may include inability to urinate, loss of appetite and unexplained weight loss, feeling tired or weak (fatigue), and bone pain.

BLCA is often first suspected through hematuria, with diagnosis confirmed by cystoscopy, ultrasound, or CT urography. About 70% of cases are detected at early stages, typically treated with transurethral resection, sometimes combined with biopsy or intravesical therapy for high-risk patients [11]. Around 30% of cases present as muscle-invasive BLCA (MIBC), for which radical cystectomy with neoadjuvant or adjuvant chemotherapy is the standard, while radiation may also be used [11]. Metastatic BLCA has a poor prognosis with a 5% five-year survival rate; platinum-based chemotherapy remains standard, though immunotherapies such as checkpoint inhibitors are emerging as promising options [11].

While survival rates have improved due to earlier detection, advancements in robotic surgical techniques, and the introduction of immunotherapy, BLCA continues to be a significant and increasing health concern globally, particularly in developed countries [1]. Consequently, there is a pressing need for a fully automated and reliable system for classifying smooth muscle images.

Recent years have seen major advances in artificial intelligence (AI) within medicine, positioning it to revolutionize practice [12]. AI’s primary advantage over clinicians is its effectiveness in utilizing diverse medical data to assist with disease diagnosis and prognosis [13]. In BLCA specifically, prior work has shown radiomics and machine learning (ML) can feasibly access pathological grade and muscle invasiveness [14]. Yet, there is limited research on identifying histological variants using radiomics, particularly CT.

This study introduces a fine-tuned EfficientNet-B3 model for BLCA detection that is designed to help clinicians identify BLCA at an earlier stage while reducing both diagnostic time and cost. Moreover, the study utilized the five-fold cross-validation method to enhance the accuracy of the EfficientNet-B3 model. This five-fold cross-validation enabled effective adjustment of the model parameters. The five-fold cross-validation is just a specific instance of k-fold cross-validation, where k can be any integer greater than 1 (common values are 3, 5, 10). The principles remain the same: the data is split into k folds, and the process is repeated k times, with each fold serving as the validation set once. The five-fold cross-validation is a powerful and widely used technique that helps in getting a more accurate and reliable assessment of DL model’s performance on unseen data. We evaluated this model on the EBTC dataset in a multiclass classification setting, first applying resizing and normalization methods to standardize the input images. The dataset was then partitioned into 80% for training and 20% for testing. In-depth experiments were carried out utilizing the EBTC dataset, along with ablation studies to determine the best hyperparameters. A thorough statistical analysis and comparisons with five leading DL models—ConvNeXtBase, DenseNet-169, MobileNet, ResNet-101, and VGG-16—showed that the proposed model outperformed the others. A summary of our research contributions is outlined below:

- A highly optimized classifier, fine-tuned from the EfficientNet-B3 architecture, was introduced to detect BLCA using the EBTC dataset.
- The EfficientNet-B3 model was combined with a five-fold cross-validation method to effectively adjust the model parameters and improve its accuracy.
- The proposed model, combined the five-fold cross-validation method with EfficientNet-B3, was carefully designed to maintain a balanced performance and reduce problems associated with overfitting.
- Our model demonstrated exceptional accuracy in identifying BLCA while utilizing minimal time and resources.
- Using our proposed model from the start showed promise in rapidly identifying BLCA, demonstrating its value in pathology by facilitating timely and personalized patient care.
- An ablation study was conducted to validate design choices made during model development.
- A comprehensive statistical comparison across all evaluated models was performed.
- When benchmarked against five DL architectures (ConvNeXtBase, DenseNet-169, MobileNet, ResNet-101, and VGG-16), the proposed approach excelled in multi-class classification, achieving 99.03% accuracy, 99.30% specificity, 97.95% precision, 96.85% recall, and a 97.36% F1-score.

The rest of this paper is organized as follows: Section 2 reviews existing literature on BLCA diagnostic systems. Section 3 describes the EBTC dataset and outlines our methodology. Section 4 presents the experimental results of the proposed framework. Finally, Section 5 concludes with a summary of our findings.

## 2. Literature Review

The diagnosis of BLCA is a prominent area of research within medical image analysis. Numerous studies approach this issue from various angles. For example, Lazo, J.F. et al. [15] introduced a method based on a semisupervised Generative Adversarial Network (GAN) that included three primary components: a teacher network trained on labeled WLI data, a cycle-consistency GAN for unpaired image-to-image translation, and a multi-input student network. To assess the quality of the synthetic images produced by the GAN, authors conducted a comprehensive quantitative and qualitative analysis with the assistance of specialists. The proposed method achieved average classification metrics for tissue classification with an accuracy of 0.90, precision of 0.88, and recall of 0.89. In contrast, the same metrics in the unlabeled domain (NBI) are 0.92 for accuracy, 0.64 for precision, and 0.94 for recall.

Yildirim, M. [16] introduced two systems centered around classification and Content-Based Image Retrieval (CBIR). The main objective of CBIR systems was to assess the visual similarities between an image provided by the user and the images stored in the database, returning those that are most alike. In the developed CBIR system, five distinct CNNs, two varying methods for textural feature extraction, and seven different metrics for measuring similarity were evaluated for feature selection and similarity assessment. The effective feature extraction techniques and similarity metrics established the foundation of the system. Densenet201 was selected for feature extraction within the system. Among the seven metrics tested, the cosine metric proved to be the most effective for measuring similarity in the proposed CBIR system. The findings indicated that the proposed CBIR model achieved 99.0% accuracy across 4 classes (High-Grade Carcinoma (HGC), Low-Grade Carcinoma (LGC), Non-Superficial Tumor (NST), and Non-Tumor Lesion (NTL) by employing the Densenet201 model for feature extraction combined with the Cosine similarity measurement method.

Sunnetci, K.M. et al. [17] assessed the performance of three proposed models using the EBTC dataset. The first model is a DL network based on CNNs. The second model, referred to as hybrid CNN-ML or DL + ML, combined the classification of deep features extracted from the CNN with ML techniques. The third model, which exhibited the highest performance metrics, was based on a vision transformer (ViT) architecture. Additionally, a graphical user interface (GUI) was provided to facilitate an accessible decision support system. The resulting accuracy and F1 score values for the DL, DL + ML, and ViT models were 0.9086, 0.8971, and 0.9257, and 0.8884, 0.8496, and 0.8931, respectively.

Sharma, P. et al. [18] introduced a hybrid model that combined CNN with a less attention-focused ViT for diagnosing bladder lesions. The model featured two inceptionV3 blocks to extract spatial characteristics. Additionally, it employed hybrid attention modules integrated into the ViT encoder to capture global correlations among the features. In tests conducted on a dataset of 17,540 endoscopic images, the model attained average metrics of 97.73% accuracy, 97.21% precision, and 96.86% F1-score through a 5-fold cross-validation approach.

Kaushik, P. et al. [19] introduced a CNN model developed and assessed for distinguishing between the tissue types using a dataset of 168 images. The findings indicated that the model was highly effective, achieving an overall accuracy of 80%. According to the classification report, HGC and NST demonstrated strong performance, both achieving a precision of 0.95. LGC also performed well, with a precision of 0.88 and a recall of 0.82. The model achieved perfect recall for NTL, although it faced challenges with precision, highlighting a precision-recall tradeoff. The macro average metrics reflected a precision of 0.77, a recall of 0.88, and an F1-score of 0.77. In contrast, the weighted averages indicated a robust performance, featuring a precision of 0.89 and an F1-score of 0.82.

Lutviana, L. et al. [20] created a CNN model for classifying bladder tissue lesions using endoscopic images. The dataset utilized comprises 1754 images categorized into four distinct classes: HGC, LGC, NST, and NTL. The CNN model developed achieved a validation accuracy of 96.29%, demonstrating strong recall, precision, and F1-scores across most classes.

The limitations of the previous studies are as follows:

1. The researchers in the earlier study focused on metrics like accuracy, precision, recall, and F1-score; however, they overlooked the evaluation of results using statistical confidence intervals (CIs). In contrast, we have effectively utilized CIs to analyze and compare results from the six different DL models. This underscores the unique advantage of our approach.
2. The authors of the previously cited research did not perform an ablation study. In contrast, we carried out this investigation to determine how each element or feature of our proposed model influences performance, systematically modifying or removing components and assessing the resulting effects.

## 3. Research Methods and Materials

### 3.1. EBTC Dataset

The proposed DL model was evaluated using the EBTC dataset [21]. This dataset was utilized in the article titled “Semi-Supervised Bladder Tissue Classification in Multi-Domain Endoscopic Images” [15]. As shown in Table 1, it consists of 1754 endoscopic images collected from 23 patients who underwent Trans-Urethral Resection of Bladder Tumor (TURBT). The images were labeled based on the histopathological analysis of the resected tissues. Typically, this endoscopic procedure was performed using White Light Imaging (WLI), and Narrow Band Imaging (NBI) was also employed when available. Four distinct categories of BLCA have been established based on the classifications provided by the World Health Organization (WHO) and the International Society of Urological Pathology. For cancerous tissues, two classifications were recognized: LGC and HGC. Furthermore, two additional categories were designated for NTL, which include cystitis resulting from infections or other inflammatory causes, as well as NST.

### 3.2. Methodology

**Figure 1:**
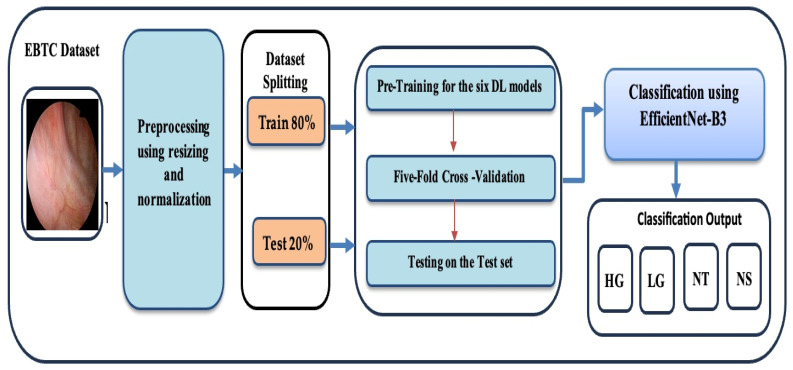
The proposed Workflow for the training and evaluating the six DL models.

To diagnose BLCA using endoscopic images, we introduced a DL model designed for multiclass classification of BLCA, utilizing the EBTC dataset. This DL model is based on EfficientNet-B3. The performance of the model was evaluated using the EBTC dataset for multiclass classification. The architecture of the proposed DL model is illustrated in Figure 1. The fine-tuning process for the six DL models is detailed in Algorithm 1. The steps involved in the proposed DL model are outlined as follows:

Phase 1 (EBTC Preprocessing): During the initial stage, the EBTC dataset was acquired from Kaggle [21] and underwent preprocessing, where the endoscopic images from the EBTC dataset were rescaled and normalized.

Phase 2 (EBTC Splitting): In the second stage, 80% of the EBTC dataset (1403 images) was allocated for training, while the remaining 20% (351 images) was reserved for testing. To prevent data leakage, all data from a single patient were assigned exclusively to either the training or test set, and similarly, each patient appeared in only one-fold during cross-validation. This ensured that no patient’s data was shared across training and validation/test sets.

Phase 3 (Pre-training six DL Models): In the third stage, six cutting-edge pre-trained DL models—originally trained on the ImageNet dataset—were selected. These include EfficientNet-B3, ConvNeXtBase, DenseNet-169, MobileNet, ResNet-101, and VGG-16.

Phase 4 (Five-Fold Cross Validation): During the 4th stage, we applied the five- fold cross-validation technique. In this technique, the training dataset was evenly split into five subsets. In each round, one subset was designated as the validation set, and the other four subsets were used for training the model. Each round iteration represents a distinct training and validation process to update the model’s parameters. This procedure was repeated five times, with each subset being used once as the validation set. The average performance across all five iterations was then computed to assess the model’s generalization ability.

Phase 5 (Multi-classification): In the final stage, the six DL models were employed to perform multi-class classification on the EBTC dataset. Furthermore, the performance of our proposed DL approach was evaluated using standard evaluation metrics.
**Algorithm 1.** Six different DL models training and evaluation1**Input** 
→
 *EBTC* dataset2**Output** 
←
 Six DL model for BLCA.3
**BEGIN**
4      **STEP 1**: **Images Preprocessing**5    **FOR EACH** I **IN** the 
EBTC   
**DO**6                *Resize* I to 224 × 224.7              *Normalize* I’s pixel values from [0, 255] to [0, 1].8            **END FOR**
9      **STEP 2: EBTC Splitting**
10            **SPLIT** 
EBTC
**INTO**11                *Training set* → 80%.12                *Testing set* 
→
 
20%.
13
      **STEP 3: Six DL Pre-Training**
14            **FOR EACH** M **IN** [EfficientNet-B3, ConvNeXtBase, DenseNet-169, MobileNet, ResNet-101, and VGG-16] **DO**15                *Load* and *pre-train* M on the ImageNet dataset.16            **END FOR**
17        **STEP 4: Cross-Validation**
18            **FOR EACH** pre-trained M **IN** [EfficientNet-B3, ConvNeXtBase, DenseNet-169, MobileNet, ResNet-101, and VGG-16] **DO**19                        **FOR EACH k = 1 to 5**
20                                    *Select* four out of the five total folds, excluding the k-th fold, to serve as the training set.21                                    *Use* the k-th fold (the one left out) as the validation set.22                                    *Train* the model using the training set, then *test* its performance on the validation set (fold k).23                                    *record* the evaluation metrics and *Calculate* the average of the performance metrics for M.24                          **END FOR**
25            **END FOR**
26
**STEP 5: Classification**
27            **FOR EACH** M model **IN** [EfficientNet-B3, ConvNeXtBase, DenseNet-169, MobileNet, ResNet-101, and VGG-16] **DO**28                  *Evaluate* the performance of M model using the test portion of the EBTC dataset.29            **END FOR**
30
**END**


#### 3.2.1. Data Preprocessing

Because data preprocessing plays a crucial role in determining the performance of DL models, it was the initial step in our experimental pipeline. We specifically focused on standardizing the endoscopic images from the EBTC dataset. This involved two key operations:

- Resizing: All images were uniformly resized to 224 × 224 pixels, which is a common input dimension for many CNNs, ensuring compatibility with pretrained models and reducing computational complexity.
- Normalization: The pixel intensity values, originally ranging from 0 to 255, were scaled to a [0, 1] range. This transformation helps stabilize the training process and accelerates convergence by reducing internal covariate shift.

By performing these preprocessing steps, we ensured that the input data was consistent and suitable for efficient model training and evaluation.

#### 3.2.2. EfficientNet-B3

EfficientNet was introduced by Tan and Le (2019) from Google AI in the paper titled “EfficientNet: Rethinking Model Scaling for Convolutional Neural Networks” [22]. The EfficientNet family, which includes models B0 through B7, was developed using a novel compound scaling method. This method uniformly scales the depth, width, and resolution of convolutional networks in a principled manner, resulting in significant improvements in both accuracy and efficiency. EfficientNet-B3 is a mid-sized model within the EfficientNet family, providing a balanced trade-off between computational cost and accuracy. It builds upon a baseline architecture known as MBConv (Mobile Inverted Bottleneck Convolution) and employs depthwise separable convolutions to enhance parameter efficiency. The scaling of EfficientNet-B3 from the base EfficientNet-B0 model includes: Width multiplier: 1.2×, Depth multiplier: 1.4×, and Input resolution: 300 × 300 pixels.

With approximately 12 million parameters, EfficientNet-B3 is smaller and faster than ResNet and Inception models that offer similar accuracy. It achieves high accuracy with fewer parameters compared to conventional CNNs (like ResNet and VGG) and enables faster training and inference through efficient computational resource usage. Additionally, it generalizes better across diverse datasets and tasks, making it particularly well-suited for medical imaging, where data volume may be limited but high precision is essential. However, it requires careful image preprocessing, such as specific input sizes. EfficientNet-B3 may underperform on non-natural images without fine-tuning, and training from scratch can be computationally intensive, which is why transfer learning is typically employed. Its interpretability is also limited compared to classical machine learning or simpler CNNs [22].

**Figure 2:**
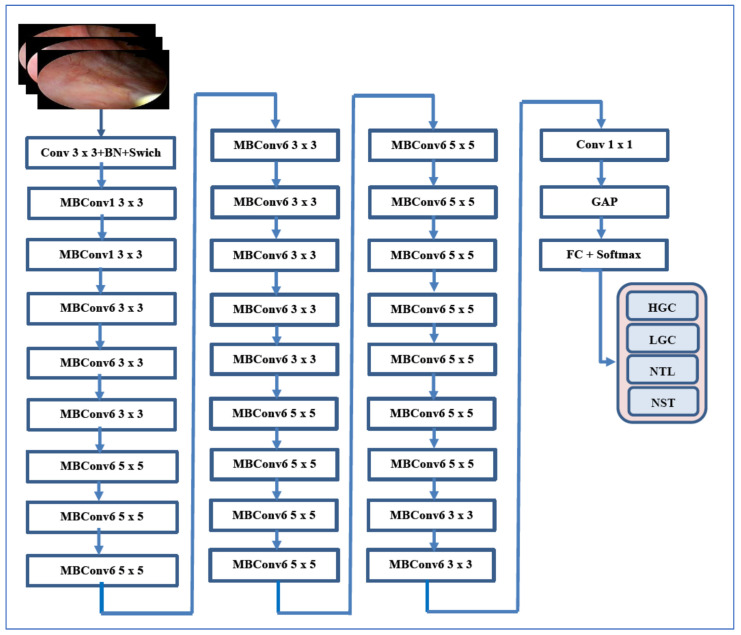
The EfficientNet-B3’s architecture for automated BLCA detection and classification.

EfficientNet-B3 has been successfully utilized in endoscopic bladder tissue classification, especially with the public EBTC dataset. Studies have employed EfficientNet-B3 for feature extraction, followed by traditional classifiers (like SVM and KNN), or have fine-tuned it end-to-end on histopathological and cystoscopic images. Its capability to capture subtle textural differences between healthy and cancerous tissue—particularly in Narrow Band Imaging (NBI) and White Light Imaging (WLI) endoscopy—makes it highly effective for detecting both low- and high-grade bladder carcinoma. Figure 2 for an illustration of EfficientNet-B3’s architecture [22].

#### 3.2.3. Five DL Techniques

In this study, five additional DL models were employed to assess the experimental results. These models are VGG-16 [23], ConvNeXtBase [24], MobileNet [25], ResNet-101 [26], and DenseNet-169 [27]. Their comparative analysis is presented in Table 2. Table 2 outlines the architectural distinctions between EfficientNet-B3 and the other models.

## 4. Evaluation and Analysis

### 4.1. Evaluated Performance Metrics

The effectiveness of the six DL models—EfficientNet-B3, ConvNeXtBase, DenseNet-169, MobileNet, ResNet-101, and VGG-16—was evaluated using the formulas presented in Equations (1)–(7).

(1) $$Accuracy=\frac{\left(TP+TN\right)}{\left(TP+FP+TN+FN\right)}$$

(2) $$Precision=\frac{TP}{\left(TP+FP\right)}$$

(3) $$Sensivity=\frac{TP}{\left(TP+FN\right)}$$

(4) $$Specifity=\frac{TN}{\left(TN+FP\right)}$$

(5) $$F1-score=\frac{2\times Precision\times Recall}{Precision+Recall}$$

(6) $$False\;Negative\;Rate\;(FNR)=\frac{FN}{TP+FN}$$

(7) $$Negative\;Predictive\;Value\;(NPV)=\frac{TN}{TN+FN}$$

True Positive (*TP*) indicates that the model correctly predicts positive cases, and this prediction is valid (positive) in the dataset. True Negative (*TN*) indicates that the model correctly predicts negative cases, and this prediction is valid (negative) in the dataset. False Positive (*FP*) indicates that the model incorrectly predicts a positive case, but the actual result is negative (no tumor) in the dataset. False Negative (*FN*) indicates that the model incorrectly predicts a negative case, while the actual result is positive (has tumor) in the dataset. *NPV* indicates the ratio of *TN* predictions, which represent normal patients. FNR indicates the ratio of *FN*. The sum of *TP* and *FN* represents the total number of patients with tumors.

### 4.2. Evaluation Environment

In this study, we carried out one experiment to evaluate the classification performance of our model on the EBTC dataset. The experiment was run in an environment powered by an AMD Ryzen 9 7950X processor (Advanced Micro Devices, Inc. (AMD), Santa Clara, CA, USA) (4.5 GHz) and 32 GB of RAM. For implementation, we utilized Python 3 in conjunction with TensorFlow, a DL framework developed by Google. TensorFlow is extensively used in AI and ML applications due to its flexibility and support for neural network development and deployment. Moreover, the specific hyperparameters applied in the experiment—such as learning rate, batch size, number of epochs, and optimizer type—are detailed in Table 3.

### 4.3. Model Performance Evaluation

The primary goal of our experiment was to identify BLCA, aiming to improve patient outcomes, simplify the diagnostic process, and significantly reduce both patient time and costs. In the experiment, 80% of the EBTC dataset, consisting of 1403 images, was designated for training, while the remaining 20% (351 images) was set aside for testing. We employed supervised pre-training to pre-train six DL models—EfficientNet-B3, ConvNeXtBase, DenseNet-169, MobileNet, ResNet-101, and VGG-16—where the six DL models were trained on the ImageNet dataset.

Additionally, in our experiment, we utilized the five-fold cross-validation technique. This method involved dividing the training dataset into five equal subsets. To prevent data leakage, all data from a single patient were assigned exclusively to either the training or test set, and similarly, each patient appeared in only one-fold during cross-validation. This ensured that no patient’s data was shared across training and validation/test sets.

In each iteration, one subset was used as the validation set while the remaining four subsets were utilized for training the model. Each iteration represented a unique training and validation process that updated the model’s parameters. This procedure was repeated five times, with each subset serving as the validation set once. The average performance across all five iterations was calculated to evaluate the model’s generalization ability. At the conclusion of the experiment, we applied the measured metrics (Equations (1)–(7)) to the six DL models.

**Figure 3:**
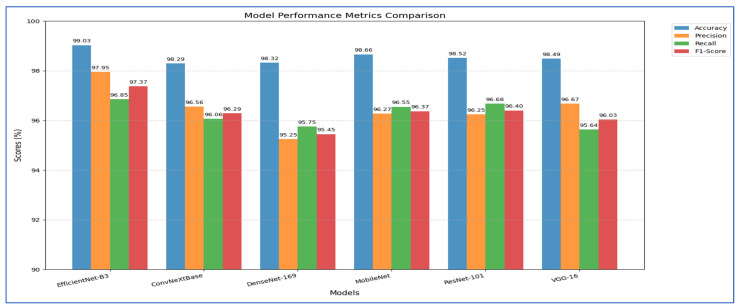
Measured Metrics for the Six DL Models.

The outcomes of the five-fold cross-validation process for the six DL models, along with the evaluation metrics, are detailed in Table 4, Table 5, Table 6, Table 7, Table 8 and Table 9 and Figure 3. The average accuracy results from the five-fold cross-validation process for the models were as follows: EfficientNet-B3 achieved an accuracy rate of 99.03%, ConvNeXtBase reached 98.29%, DenseNet-169 attained 98.32%, MobileNet recorded 79.09%, and VGG-16 achieved 98.49%. Based on these results, EfficientNet-B3 demonstrated the highest accuracy among the models evaluated.

**Figure 4:**
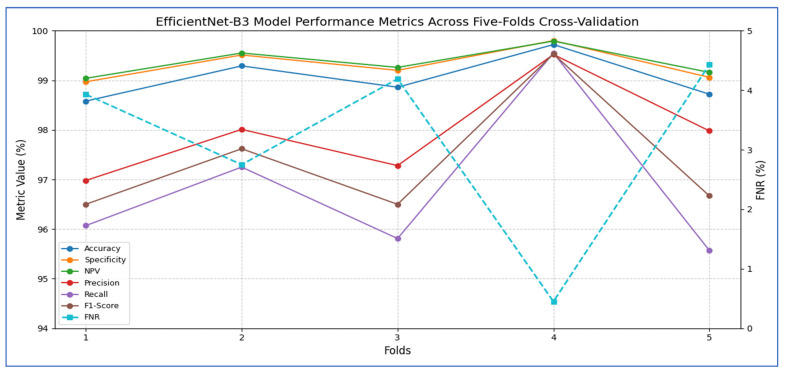
EfficientNet-B3 model performance metrics across five-fold cross-validation.

Table 4 and Figure 4 show that the EfficientNet-B3 model was assessed using five cross-validation folds, with the following performance metrics reported:

1. Accuracy and Specificity
  ○ The model’s accuracy ranged from 98.58% to 99.72% across the five folds, yielding an overall mean accuracy of 99.03%.
  ○ The specificity (true-negative rate) was consistently high, fluctuating between 98.97% and 99.80%, and averaging 99.31%.
  ○ These results indicate that the model rarely misclassified negative cases, demonstrating a strong ability to accurately identify healthy samples.
2. FNR and NPV
  ○ The FNR (the proportion of actual positives missed) ranged from 0.45% (fold 4) to 4.43% (fold 5), with an average of 3.15%.
  ○ The corresponding NPV (the probability that a negative prediction was truly negative) varied between 99.04% and 99.79%, averaging 99.36%.
  ○ This indicates that when the network predicted a negative class, it was almost always correct.
3. Precision, Recall, and F1-Score
  ○ Precision (positive predictive value) ranged from 96.98% to 99.52%, with a mean of 97.95%, indicating that most of the model’s positive predictions were true positives.
  ○ Recall (sensitivity) showed wider variability, ranging from 95.57% (fold 5) to 99.55% (fold 4), averaging 96.85%.
  ○ The F1-score, which balances precision and recall, spanned from 96.50% to 99.53%, with an overall mean of 97.37%.

Overall, the EfficientNet-B3 model demonstrated extremely high specificity and precision, a low false-negative rate, and very strong accuracy. Slight variability in recall across folds suggests occasional missed positive instances, but the model’s average F_1_-score above 97% reflects an excellent balance between sensitivity and precision.

**Figure 5:**
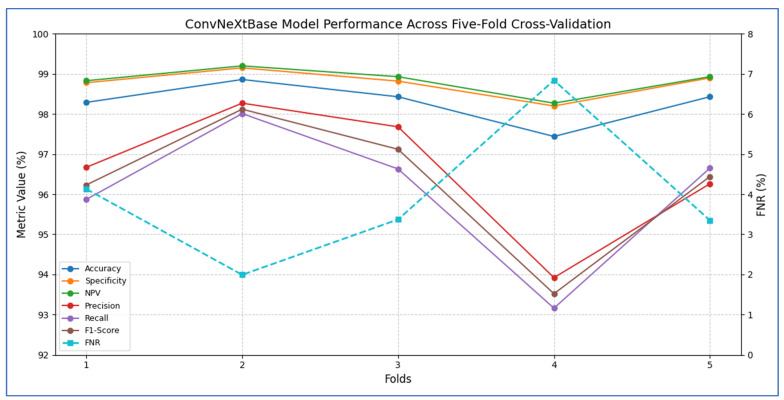
ConvNeXtBase model’s performance metrics across five-fold cross-validation.

Table 5 and Figure 5 present that the average accuracy of the ConvNeXtBase reached 98.29%, demonstrating the model’s overall reliability in classification tasks. Specificity was also high across all folds, averaging 98.77%, indicating effective identification of true negative cases. The FNR averaged 3.94%, indicating a low likelihood of missing positive cases, which is vital in clinical or sensitive contexts. The NPV was strong at 98.83%, reflecting high confidence in negative predictions. Regarding precision, the model achieved an average of 96.56%, showcasing a low rate of FPs. The recall, measuring the model’s ability to detect all actual positive cases, averaged 96.06%, indicating general effectiveness in identifying positive instances. The F1-score, representing the harmonic mean of precision and recall, stood at 96.29%, confirming the model’s balanced performance.

In terms of fold-wise performance, Fold 2 excelled across most metrics, particularly in precision (98.27%), recall (98.01%), and F1-score (98.12%). In contrast, Fold 4 exhibited the lowest performance, with accuracy (97.44%), recall (93.16%), and F1-score (93.52%), suggesting some variability in model generalization based on data splits. Overall, the ConvNeXtBase model demonstrated excellent and stable performance across different folds, with only minor fluctuations in metrics.

**Figure 6:**
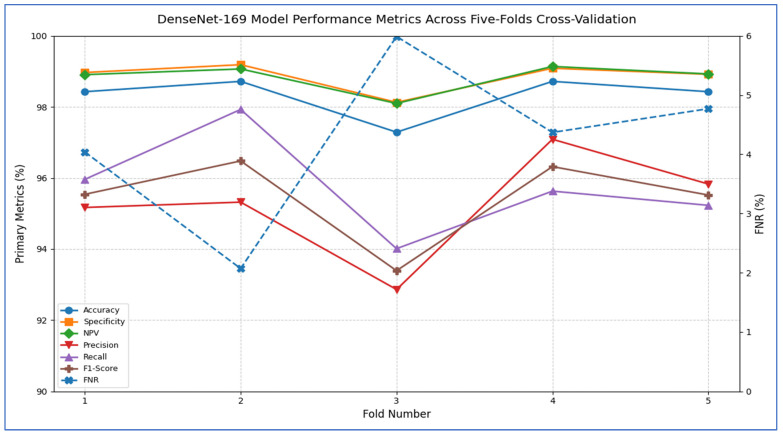
DenseNet-169 model’s performance metrics across five-fold cross-validation.

The results in Table 6 and Figure 6 showed that the ConvNeXtBase model demonstrated consistently strong performance across all five folds. Fold 2 achieved the highest metrics with an accuracy of 98.86%, specificity of 99.15%, NPV of 99.20%, precision of 98.27%, recall of 98.01%, and F1-score of 98.12%. In contrast, Fold 4 recorded the lowest values: 97.44% accuracy, 98.20% specificity, 98.27% NPV, 93.92% precision, 93.16% recall, and 93.52% F1-score. The FNR varied from a low of 1.99% in Fold 2 to a high of 6.84% in Fold 4, indicating greater class imbalance or more challenging cases in that split. On average, the model achieved: accuracy: 98.29%; specificity: 98.77%; FNR: 3.94%; NPV: 98.83%; precision: 96.56%; recall: 96.06%; F1-score: 96.29%.

These averages suggest that ConvNeXtBase was highly effective at accurately identifying both positive and negative cases, with particularly strong specificity and NPV reflecting a very low rate of false positives. The slightly lower average recall and F1-score, influenced mainly by Fold 4, indicated that a small proportion of true positive cases were missed in the most difficult partition. Nevertheless, the overall performance of the model remained robust and well balanced.

**Figure 7:**
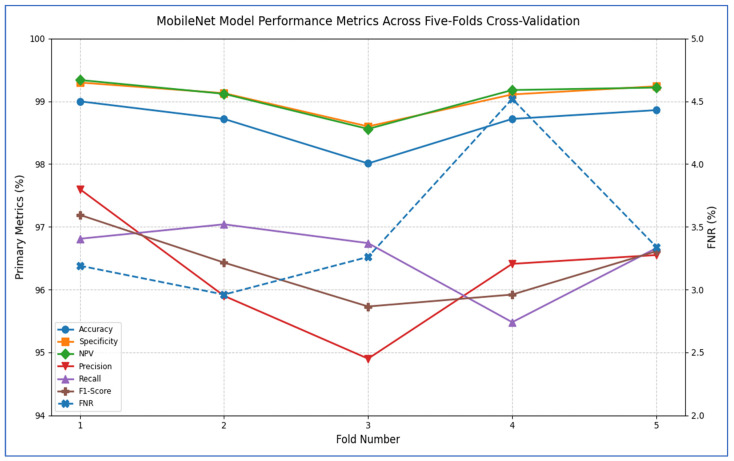
MobileNet model’s performance metrics across five-fold cross-validation.

Table 7 and Figure 7 show that the MobileNet model showed consistently strong performance across all five cross-validation folds. Accuracy ranged from 98.01% (fold 3) to 99.00% (fold 1), resulting in an average accuracy of 98.66%. Specificity was also high, varying between 98.60% and 99.30%, with a mean of 99.08%. The FNR was low overall, ranging from 2.96% to 4.52% (mean 3.45%), indicating that the model rarely missed positive cases. Correspondingly, the NPV remained above 98.56% for every fold, averaging 99.08%, which reflects strong confidence in negative predictions. On the positive side, precision varied from 94.90% (fold 3) to 97.60% (fold 1), averaging 96.27%. Recall (sensitivity) ranged from 95.48% to 97.04%, with a mean of 96.55%. The resulting F1-scores ranged between 95.73% and 97.19%, averaging 96.37%, signifying that the model effectively balanced precision and recall. Overall, MobileNet maintained high discriminatory power (average specificity 99.08%) and robust detection capability (average recall 96.55%), with very few false negatives, underscoring its reliability for this classification task.

**Figure 8:**
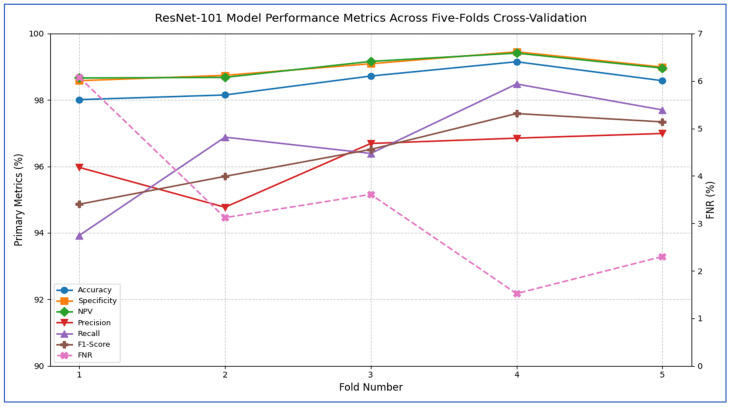
ResNet-101 model’s performance metrics across five-fold cross-validation.

Table 8 and Figure 8 report that the ResNet-101 model demonstrated strong performance across all five cross-validation folds. Its accuracy varied from 98.01% in Fold 1 to 99.15% in Fold 4, resulting in an average accuracy of 98.52%. Specificity was consistently high, ranging between 98.58% and 99.45% (mean = 98.97%), indicating that the network reliably identified negative cases with very few false positives. The FNR reached as low as 1.52% in Fold 4 and never exceeded 6.08%, averaging only 3.32% across the folds—showing that the model rarely missed positive instances. The NPV remained above 98.66% in every fold (mean = 98.98%), confirming that when the model predicted a negative outcome, it was almost always correct. Although precision dropped to 94.77% in Fold 2, it stayed above 95% in the other folds (mean = 96.25%), indicating that most positive predictions were true positives. Recall (sensitivity) was also strong, ranging from 93.92% to 98.48% (mean = 96.68%), reflecting the model’s ability to detect actual positives. Finally, the F1-score—a harmonic mean of precision and recall—varied from 94.86% to 97.59%, averaging 96.40%, confirming a balanced trade-off between these two metrics throughout the validation process.

**Figure 9:**
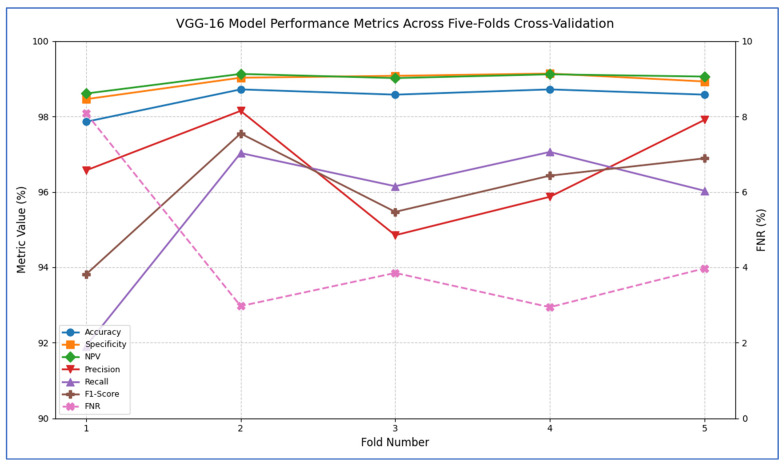
VGG-16 model’s performance metrics across five-fold cross-validation.

Table 9 and Figure 9 record that the VGG-16 model demonstrated consistently high performance across all five cross-validation folds. The accuracy ranged from 97.86% in Fold 1 to 98.72% in Folds 2 and 4, yielding an overall mean accuracy of 98.49%. Specificity was similarly robust, varying only between 98.46% and 99.14% (mean = 98.93%), which indicated that the model very reliably identified negative cases. The FNR was low in every fold (2.94–8.09%), with an average of 4.36%, reflecting that only a small proportion of positive cases were missed. Correspondingly, the NPV remained near perfection (mean = 98.99%), confirming that predictions of “negative” were almost always correct. In terms of positive-case detection, precision values spanned from 94.85% (Fold 3) to 98.15% (Fold 2), averaging 96.67%. Recall (sensitivity) was equally strong, ranging from 91.91% to 97.06% (mean = 95.64%), demonstrating that the model captured the vast majority of true positives. The resulting F_1_-scores (harmonic mean of precision and recall) fell between 93.81% and 97.55%, with an average of 96.03%, underscoring a well-balanced trade-off between precision and recall. Overall, these past results showed that VGG-16 achieved excellent discrimination ability, maintained low error rates, and delivered highly reliable positive and negative predictions across all folds.

**Figure 10:**
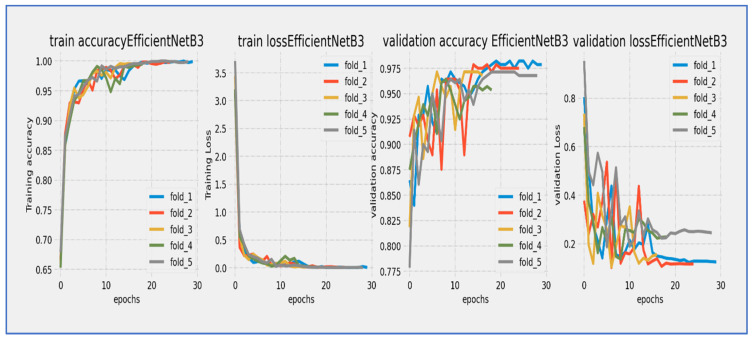
Training and validation loss and accuracy of the EfficientNet-B3 through the five-fold cross-validation.

**Figure 11:**
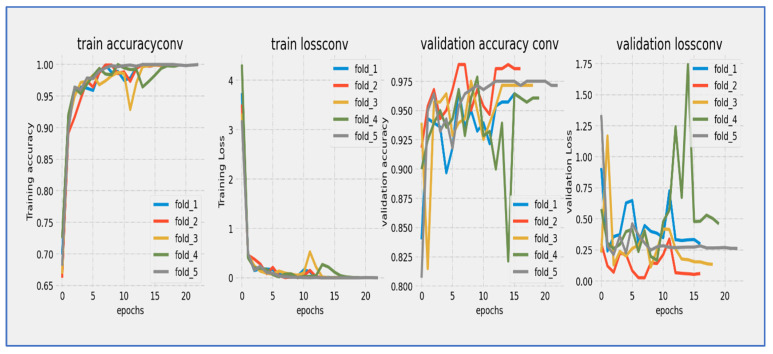
Training and validation loss and accuracy of the ConvNeXtBase through the five-fold cross-validation.

**Figure 12:**
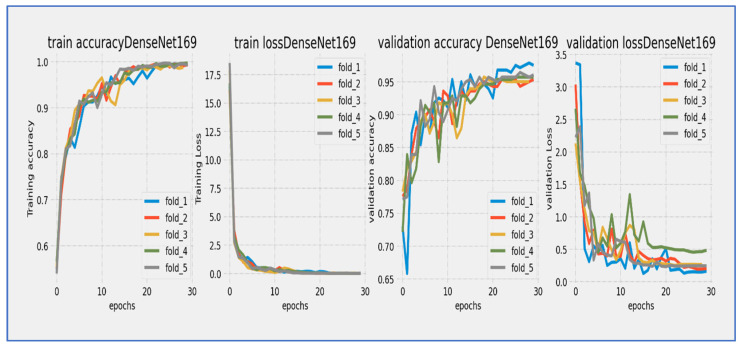
Training and validation loss and accuracy of the DenseNet-169 through the five-fold cross-validation.

**Figure 13:**
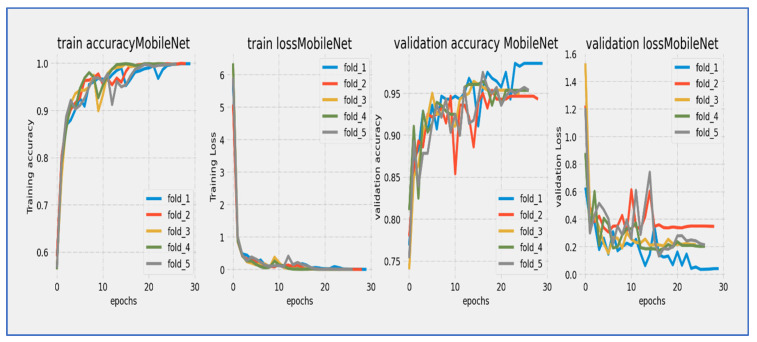
Training and validation loss and accuracy of MobileNet through the five-fold cross-validation.

**Figure 14:**
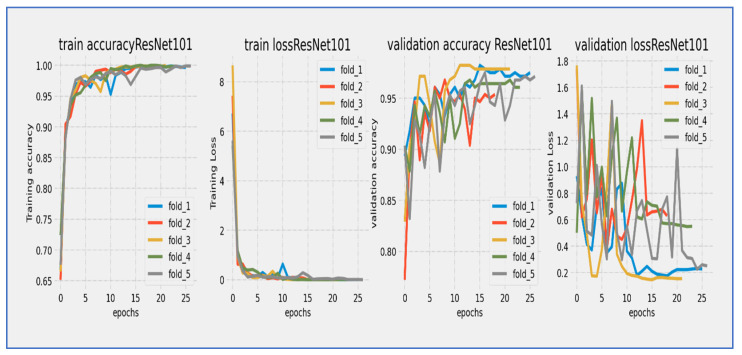
Training and validation loss and accuracy of the ResNet-101 through the five-fold cross-validation.

**Figure 15:**
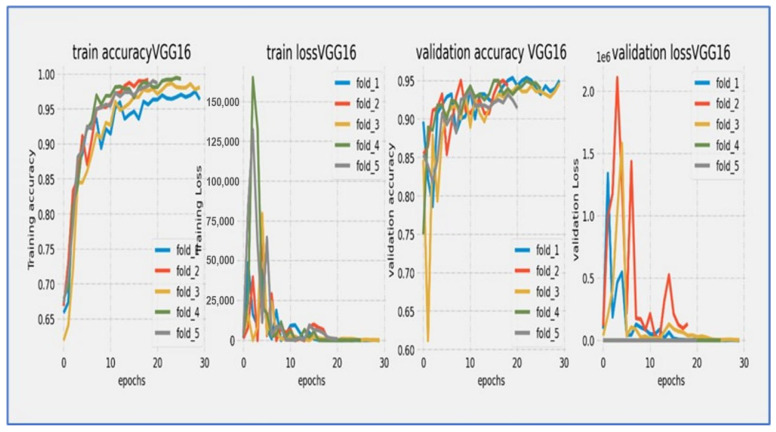
Training and validation loss and accuracy of the VGG-16 through the five-fold cross-validation.

Figure 10, Figure 11, Figure 12, Figure 13, Figure 14 and Figure 15 illustrate the training and validation loss for six DL techniques: EfficientNet-B3, ConvNeXtBase, DenseNet-169, MobileNet, ResNet-101, and VGG-16. From Figure 10, the EfficientNet-B3 model achieved successful convergence during the 25-epoch training period, with both training and validation losses consistently decreasing throughout the process. The most significant improvements were observed in the first 10 epochs.

At the start, the training loss was approximately 4.0 (epoch 0) with a steep decline noted during the initial epochs (0–5). After 15 epochs, the loss stabilized around 1.0, and by epoch 25, the final training loss reached approximately 0.5. The validation loss began slightly higher than the training loss (around 4.0+) and followed a similar decreasing trend. A small but consistent gap remained between the training and validation curves, with the final validation loss settling around 1.0 at epoch 25, without any significant divergence between the curves.

The parallel downward trend indicated good generalization, and the consistent gap of approximately 0.5 suggested mild but acceptable overfitting. The most rapid improvements occurred between epochs 0–10, with a noticeable slowdown in loss reduction after epoch 15. The model achieved its lowest loss values in the final five epochs, exhibiting stable and consistent learning behavior. The validation loss maintained a reasonable correlation with the training loss, indicating successful training and good convergence.

No signs of underfitting or severe overfitting were detected. The loss scale (0.5–4.0) was typical for well-initialized models, and the convergence pattern aligned with expectations for EfficientNet architectures. The 25-epoch duration proved sufficient for near-complete convergence. Overall, the EfficientNet-B3 model completed training with successful convergence, demonstrating a characteristic learning curve marked by steady loss reduction and effective learning, with final loss values indicating a stable and well-performing state by the end of the training cycle.

The EfficientNet-B3 model showed impressive learning capabilities over its 25-epoch training cycle. Training accuracy started at around 75% and displayed rapid, near-linear improvement during the first 10 epochs, exceeding 90% accuracy by epoch 10. Validation accuracy began at a slightly lower value (~73%) but closely followed the training curve, maintaining a consistent 2–3% gap throughout the training period. Both curves moved in stable parallel alignment after epoch 5, with no significant divergence noted. The most substantial gains happened between epochs 0–15, after which the rate of improvement slowed as the model approached its performance ceiling. By epoch 25, training accuracy leveled off at approximately 94–95%, while validation accuracy stabilized at 92–93%. The minimal and consistent gap between the curves suggested excellent generalization with negligible overfitting. The model reached its target validation accuracy benchmark (90%) by epoch 12 and continued to improve consistently through the rest of the training. The smooth, stable progression of both accuracy curves reflected well-tuned hyperparameters and effective learning dynamics characteristic of the EfficientNet architecture. 

From Figure 11, the ConvNeXtBase model showed effective learning behavior over its 20-epoch training cycle. Training loss started at approximately 4.0 and experienced a sharp, consistent decline during the initial phase, reaching about 1.0 by epoch 10. Validation loss began at a similar starting point but exhibited slightly more fluctuation, particularly between epochs 5–15, where it maintained a consistent gap of 0.3–0.5 above the training loss. Both curves progressed in parallel after epoch 15, converging to approximately 0.5–0.7 by the final epoch. The model achieved its most significant loss reduction in the first 10 epochs, after which improvements slowed considerably. While a persistent but narrowing gap existed between training and validation losses throughout the training, no concerning divergence occurred, indicating the model generalized reasonably well without severe overfitting. The final convergence pattern suggested that the 20-epoch duration was sufficient for this architecture to approach stability.

The ConvNeXtBase model showed strong learning behavior during its 20-epoch training cycle. Training accuracy started at about 75% and quickly improved in the early stages, exceeding 85% by epoch 5. Validation accuracy began at a slightly lower level (~72%) but followed the training curve closely. After epoch 5, both metrics maintained a consistent parallel trajectory, with validation accuracy lagging behind training accuracy by a steady 2–4% margin throughout the training period. The most significant gains happened between epochs 0–10, where accuracy increased at an almost linear rate of ~2% per epoch. After epoch 15, the rate of improvement slowed considerably as the model approached its performance limit. By the final epoch, training accuracy settled at approximately 94–95%, while validation accuracy stabilized at 91–92%. The model reached the 90% validation benchmark by epoch 12 and continued to show small gains in the following epochs. The persistent but narrow accuracy gap suggested mild overfitting, although the parallel curve alignment indicated that this did not significantly affect generalization capabilities.

From Figure 12, the DenseNet-169 model underwent a thorough 30-epoch training cycle, during which both training and validation losses showed characteristic convergence behavior. The training loss started at a relatively high value and displayed a steep, consistent decline throughout the first 15 epochs, gradually stabilizing thereafter. The validation loss began slightly higher than the training loss and followed a similar downward path, although with slightly greater fluctuation, especially during the mid-training phase (epochs 10–20). A noticeable but moderate gap persisted between the two curves throughout the entire training period, narrowing to approximately 0.3–0.5 units by epoch 30. The most significant loss reduction occurred in the first 20 epochs, after which both curves progressed in near-parallel alignment, indicating stabilization. Final loss values settled around 1.0 for training and 1.3–1.5 for validation, suggesting that the extended 30-epoch duration was beneficial for this architecture’s convergence. The consistent downward trend without significant divergence reflected effective learning with no severe overfitting concerns.

The DenseNet-169 model showed steady but gradual learning progress over its 30-epoch training cycle. Training accuracy started at about 60% and increased consistently throughout the training period. Validation accuracy also began at a similar level but showed slightly more fluctuations, especially during the mid-training period (epochs 10–20). Both accuracy curves kept a consistent gap of 5–8 percentage points, with validation accuracy consistently lagging behind training accuracy. The most significant improvements happened between epochs 5–20, where training accuracy rose from around 65% to 75%. By epoch 25, training accuracy neared 80%, while validation accuracy reached about 72–74%. The model continued to show slight gains in the final epochs, although the rate of improvement slowed significantly after epoch 25. The persistent accuracy gap suggested moderate overfitting challenges, while the stabilization of both curves indicated that the model was nearing its performance ceiling. The 30-epoch duration proved essential for this architecture to achieve meaningful accuracy gains, even though the final validation accuracy remained below standard benchmarks for modern architectures.

From Figure 13, MobileNet’s architecture showed a strong learning curve throughout its 25-epoch training cycle. Training loss started at about 6.0 and had a steep, almost linear decline during the first 10 epochs, reaching around 2.0 by epoch 10. Validation loss began at a similar high value but showed more fluctuations during the early training phase, briefly spiking around epoch 5 before continuing its downward trend. Both curves moved closely together after epoch 10, with training loss keeping a steady 0.2–0.5 unit advantage over validation loss. The most significant improvements happened in the first 15 epochs, after which the rate of loss reduction slowed considerably. By epoch 25, training loss ended up at about 0.5 while validation loss settled at around 1.0. The ongoing but narrowing gap between curves suggested mild overfitting, though the similar final convergence implied that the model achieved stable generalization. The extended 25-epoch duration was advantageous for this architecture, allowing it to fully utilize its efficient design features.

MobileNet’s architecture showed strong learning capabilities during its 25-epoch training cycle, although with noticeable early fluctuations. Training accuracy started at around 60% and demonstrated rapid, near-linear improvement during the first 10 epochs, exceeding 70% by epoch 5 and reaching 80% by epoch 15. Validation accuracy began at a similar level but experienced significant variations in the early training phase (epochs 0–10), including a temporary dip around epoch 5 before recovering robustly. After epoch 10, both curves moved in stable parallel alignment, with validation accuracy keeping a consistent 3–5% gap below training accuracy. The most significant gains took place between epochs 5–15, where accuracy improved at about 2% per epoch. After epoch 20, the rate of improvement slowed as the model neared its performance ceiling, with training accuracy converging to around 85–86% and validation accuracy stabilizing at 81–82% by epoch 25. The model reached the 80% validation benchmark by epoch 15 and continued to show incremental gains throughout the remaining training. The early fluctuations in validation accuracy indicated initial sensitivity to hyperparameters or data variations, while the stable late-stage convergence with a moderate gap suggested mild but manageable overfitting. The final accuracy values illustrated the efficiency of the MobileNet architecture for the given task, with validation accuracy demonstrating respectable performance despite the early fluctuations.

From Figure 14, the ResNet-101 model showed a typical learning trend over its 25-epoch training period. The training loss started near 5.0 and experienced a quick initial drop, decreasing to about 2.0 within the first 5 epochs. The validation loss began at a similar level but showed slightly more fluctuations during this early stage. Both curves followed a steady downward path throughout the training cycle, with the most significant reductions occurring before epoch 15. A consistent but moderate gap of about 0.5 to 1.0 units remained between the training and validation losses, with the validation curve staying consistently above the training curve. After epoch 15, the rate of improvement slowed significantly, with both losses moving in near-parallel alignment toward their final values. By epoch 25, the training loss reached about 0.5 while the validation loss settled around 1.5. The stable convergence pattern without late-stage divergence suggested that the model achieved effective learning with no significant overfitting issues. The 25-epoch duration proved sufficient for considerable convergence.

The ResNet-101 model showed strong learning performance during its 25-epoch training cycle. Training accuracy started at about 75% and quickly improved in the first phase, exceeding 85% by epoch 5 and reaching 90% by epoch 10. Validation accuracy began at a slightly lower level (~73%) but followed the training curve closely, maintaining a consistent 2–4% gap throughout the training period. Both metrics followed a nearly parallel path after epoch 5, showing no significant divergence. The most significant improvements happened in the first 15 epochs, with accuracy growing by about 1.5% per epoch. After epoch 15, the rate of improvement slowed as the model neared its performance ceiling, with training accuracy leveling off around 94–95% and validation accuracy stabilizing at 91–92% by epoch 25. The model reached the important 90% validation benchmark by epoch 12 and continued to show small gains in the remaining training cycles. The persistent but narrow accuracy gap suggested mild overfitting, while the absence of late-stage fluctuations indicated stable learning dynamics. The parallel movement of both curves implied that the model generalized well to validation data, with the final validation accuracy reflecting strong performance for this architecture. The 25-epoch duration proved adequate to approach the model’s maximum capability, as shown by the plateau effect in the final training phase.

From Figure 15, the VGG-16 model went through a challenging training process over about 20 epochs, as shown by its unique loss profile. Training loss started at a relatively high value near 20.0 and showed a steady downward trend throughout the training cycle. Validation loss began at a similar high level and exhibited more variability, especially during the middle training phase (epochs 5–15). A significant gap of 5–7 units remained between the training and validation curves during the first 15 epochs, narrowing slightly but still being considerable (2–4 units) by the final epochs. The most significant reduction in loss happened in the first 5 epochs, after which the rate of improvement slowed considerably. By epoch 20, training loss settled at around 5.0 while validation loss stabilized between 7.0–9.0. The consistent difference between the curves indicated notable overfitting challenges, while the relatively high final loss values suggested that the model had difficulty achieving optimal convergence within the 20-epoch cycle.

The VGG-16 model showed challenging learning dynamics during its 20-epoch training cycle. Training accuracy started at approximately 70% and steadily improved throughout the training period, progressing at a near-linear rate to reach 85% by epoch 15. Validation accuracy began at a significantly lower starting point (~65%) and diverged from the training curve. A substantial performance gap of 15–20 percentage points persisted during the early training phase (epochs 0–10), narrowing only modestly to 10–12 points by epoch 20. The validation curve displayed notable volatility, particularly between epochs 5–15, where it experienced multiple fluctuations instead of consistent improvement. While training accuracy reached 90% by epoch 17.5, validation accuracy stalled around 78–80% during the same period. The most significant gains occurred before epoch 10, after which validation accuracy showed minimal improvement despite continued training progress. By the final epoch, training accuracy converged to approximately 92–93%, while validation accuracy plateaued at 80–82%. The persistent and substantial accuracy gap indicated significant overfitting challenges, while the validation curve’s volatility suggested sensitivity to hyperparameters or data characteristics. The model did not achieve the 85% validation benchmark within the training cycle, with late-stage stagnation implying that additional epochs would yield diminishing returns. These results reflected the architectural limitations of VGG-16 for this specific task, particularly its tendency toward overfitting without extensive regularization techniques.

Hence, EfficientNet-B3, DenseNet-169, and ConvNeXtBase demonstrated the best overall performance. These architectures achieved the highest training and validation accuracies (above 94% on average) while maintaining consistently low validation losses across folds, indicating excellent generalization and stable learning. MobileNet and ResNet-101 also performed well, achieving strong accuracy levels above 92% with relatively low losses, although they exhibited slightly more fluctuations in validation performance compared to the top models. In contrast, VGG-16 showed the weakest results, with lower validation accuracy (around 85–90%) and higher, more unstable validation losses, suggesting less robust generalization and potential overfitting. Overall, EfficientNet-B3 emerged as the most reliable model due to its combination of rapid convergence, high validation accuracy, and minimal loss variations, making it the best candidate for applications requiring high precision and stability. Table 10 presents a comparative performance for the six DL techniques.

**Figure 16:**
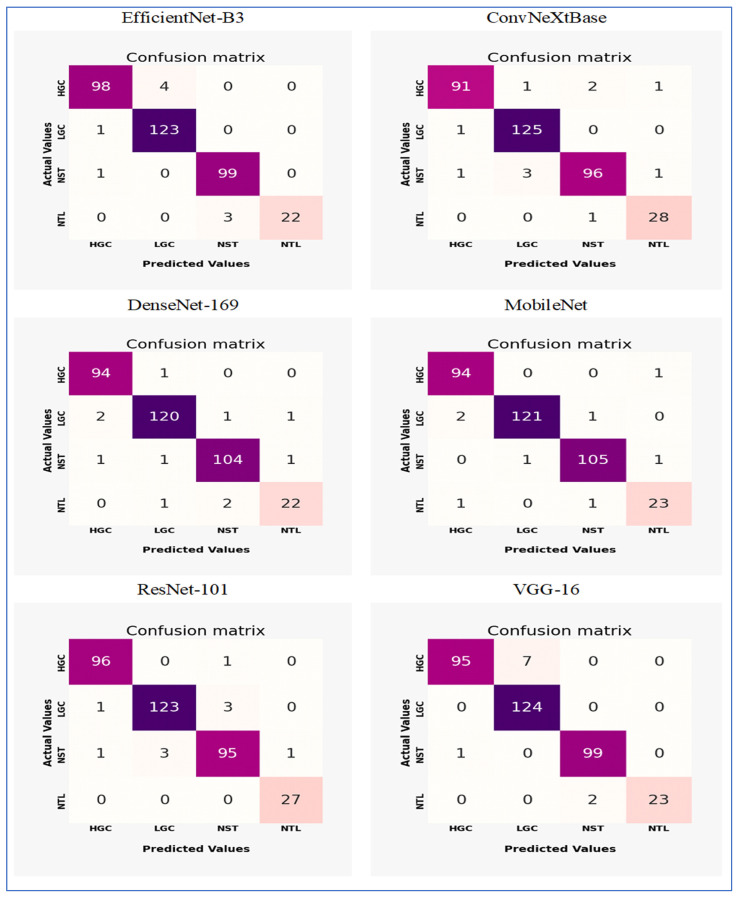
The confusion matrices for the six DL models through the five-fold cross-validation.

Figure 16 shows the confusion matrices for EfficientNet-B3, ConvNeXtBase, DenseNet-169, MobileNet, ResNet-101, and VGG-16. These models were evaluated on the test set of the EBTC dataset, which contains a total of 1403 images designated for training. The remaining 20% (351 images) were set aside for testing. The EBTC dataset includes four classes: HGC (469 images), LGC (647 images), NTL (134 images), and NST (504 images). The test set for EBTC consists of 102 images from the HGC class, 124 images from the LGC class, 25 images from the NTL class, and 100 image from the NST class.

The EfficientNet-B3 model showed strong classification performance with notable strengths and minor limitations across the four tissue classes (HGC, LGC, NST, and NTL). The model achieved exceptional precision in identifying LGC (123 correct predictions) and NST (99 correct), with near-perfect recall for NST (99/100 = 99%) and NTL (22/25 = 88%). However, it displayed moderate confusion between HGC and LGC classes, where 4 HGC samples were misclassified as LGC (FN) and 1 LGC sample was incorrectly assigned to HGC (FP). The NTL class encountered minor challenges, with 3 samples misidentified as NST, indicating potential morphological similarities between these categories. Importantly, no cross-confusion occurred between biologically distinct classes (e.g., HGC vs. NST or LGC vs. NTL), reflecting the model’s effective learning of key discriminative features. The overall accuracy reached 97.3% (342 correct predictions out of 351 total samples), although the HGC class exhibited the lowest recall (98/103 ≈ 95.1%) due to LGC misclassifications. These results highlighted the model’s proficiency while emphasizing HGC-LGC differentiation as the primary area for potential improvement.

The ConvNeXtBase demonstrated strong classification abilities with minimal confusion between the four tissue categories (HGC, LGC, NST, and NTL). The model achieved outstanding results for LGC (125 correct predictions) and NTL (28 out of 29, approximately 96.6% recall), showing near-perfect specificity for these classes. However, it encountered significant difficulties in identifying HGC, with 4 samples misclassified (1 as LGC, 2 as NST, and 1 as NTL), resulting in the lowest class recall (91 out of 95, approximately 95.8%). Similarly, the NST class experienced moderate confusion, with 5 errors (1 misassigned to HGC, 3 to LGC, and 1 to NTL), leading to a recall of 96 out of 101, approximately 95.0%. Cross-class confusion primarily involved histologically similar categories: HGC-NST misclassifications hinted at difficulties in distinguishing high-grade features, while NST-LGC errors suggested possible overlap in stromal patterns. The model maintained strong diagonal dominance with 340 correct predictions out of 351 total samples (96.9% accuracy), although the error distribution revealed opportunities to enhance feature extraction for differentiating HGC and NST. Importantly, no FPs were found for LGC against NTL, confirming effective learning of essential diagnostic boundaries.

The DenseNet-169 model showed strong classification performance with excellent precision across most tissue classes, though minor inter-class confusion remained. The architecture achieved remarkable results for HGC (94 correct predictions, 1 misclassified as LGC) and NST (104 correct, 2 errors), reflecting near-perfect recall rates of 98.9% and 99.0%, respectively. However, it faced slight challenges in distinguishing LGC and NTL categories: LGC experienced 4 misclassifications (2 as HGC, 1 as NST, 1 as NTL), while NTL encountered 3 errors (1 as LGC, 2 as NST), resulting in the lowest class recall (22/25 = 88.0%). Notably, the model preserved critical diagnostic boundaries between biologically distinct classes (HGC vs. NST/NTL and LGC vs. HGC), with no cross-confusion between these pairs. The confusion between LGC and NTL implied potential similarities in stromal presentation, while NST’s single misclassification as LGC suggested minor feature overlap in necrotic patterns. With 340 correct predictions out of 351 total samples (96.9% accuracy), the model’s performance was highly competitive, though the NTL class represented the primary opportunity for improvement. The error distribution underscored the architecture’s proficiency in high-grade cancer identification while revealing subtle challenges in lower-grade and normal tissue differentiation.

The MobileNet model demonstrated outstanding classification performance with minimal confusion between classes across the four tissue categories (HGC, LGC, NST, and NTL). The model achieved nearly perfect results for HGC (94 correct predictions, 1 misclassified as NTL) and NST (105 correct, 2 minor errors), resulting in impressive recall rates of 98.9% and 99.1%, respectively. LGC identification was particularly effective, with only 3 misclassifications (2 as HGC, 1 as NST) out of 124 samples (97.6% recall). The NTL class showed slight difficulties, with 2 errors (1 as HGC, 1 as NST) against 23 correct predictions (92.0% recall), marking the highest error rate. Importantly, the model maintained excellent diagnostic boundaries between biologically distinct categories: no FPs were recorded between LGC-NTL or HGC-NST pairs, and no confusion was found between LGC and NTL classes. The single HGC-NTL misclassification implied potential ambiguity in normal tissue boundaries, while the NST-LGC error reflected minor feature overlap in stromal patterns. With 343 correct predictions out of 351 total samples (97.7% accuracy), the performance exceeded that of other architectures in overall precision. The highly diagonal confusion matrix illustrated the model’s superior feature discrimination capabilities, with NTL differentiation remaining the only area for potential enhancement despite its already strong 92% class recall.

The ResNet-101 model demonstrated excellent classification capabilities, showing particularly strong performance in critical diagnostic categories. The architecture achieved near-perfect results for NTL (27 correct predictions, 100% recall) and HGC (96 correct, 99.0% recall), with only one HGC sample misclassified as NST. LGC identification was robust (123 correct) but showed minor confusion with NST (3 misclassifications). The NST class faced moderate challenges, with 5 errors (1 as HGC, 3 as LGC, 1 as NTL) against 95 correct predictions (95.0% recall), representing the lowest performance among the classes. Critically, the model maintained essential diagnostic boundaries: no FPs occurred between HGC-NTL or LGC-NTL pairs, and no NTL samples were misassigned to other categories. The primary confusion occurred between histologically similar LGC and NST classes (accounting for 6 out of 10 errors), suggesting feature overlap in stromal presentation. With 341 correct predictions out of 351 samples (97.2% accuracy), the overall performance was highly competitive, though the error distribution highlighted opportunities to enhance NST differentiation. The isolated HGC-NST misclassification indicated rare edge cases where high-grade and necrotic features proved ambiguous, while the perfect NTL recall underscored the model’s proficiency in identifying normal tissue boundaries.

The VGG-16 model demonstrated strong classification capabilities, achieving excellent performance in three categories but exhibiting notable challenges in identifying NTL. The architecture achieved near-perfect results for HGC (95 correct predictions, 100% recall) and LGC (124 correct, 100% recall), with no misclassifications observed in these critical diagnostic categories. NST identification was similarly robust (99 correct predictions, 99% recall), with only one sample misclassified as LGC. However, the model faced significant difficulties with NTL recognition, where 2 of 25 samples were misidentified as NST, resulting in 92% recall for this class. The primary confusion occurred between histologically similar NST and NTL categories, suggesting challenges in distinguishing necrotic patterns from normal tissue boundaries. Importantly, the model maintained perfect diagnostic boundaries between cancerous (HGC/LGC) and normal (NTL) tissue types, with no FPs in these critical categories. The overall accuracy reached 97.4% (342 correct out of 351 samples), though the NTL errors highlighted ongoing challenges in normal tissue identification that aligned with observations from the architecture’s loss curve.

### 4.4. Statistical Analysis of Experimental Results

**Figure 17:**
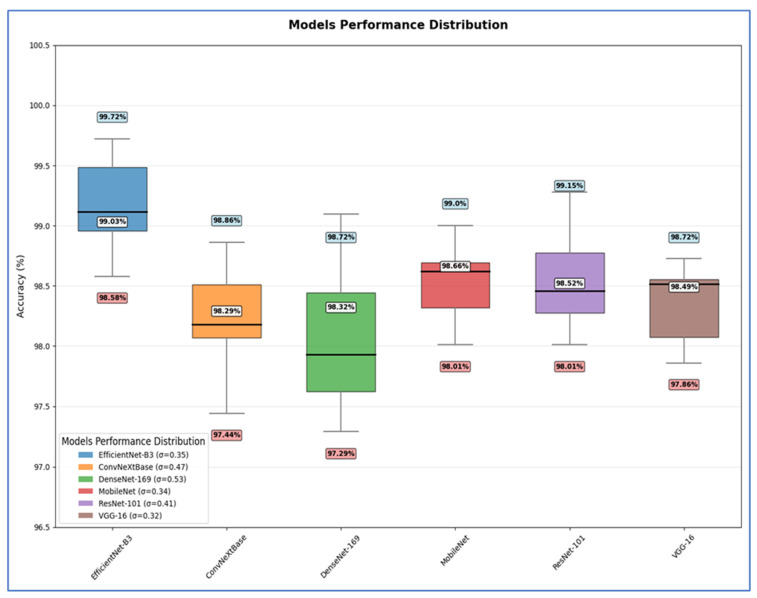
The statistical analysis of our experimental results through the five-fold cross-validation.

**Figure 18:**
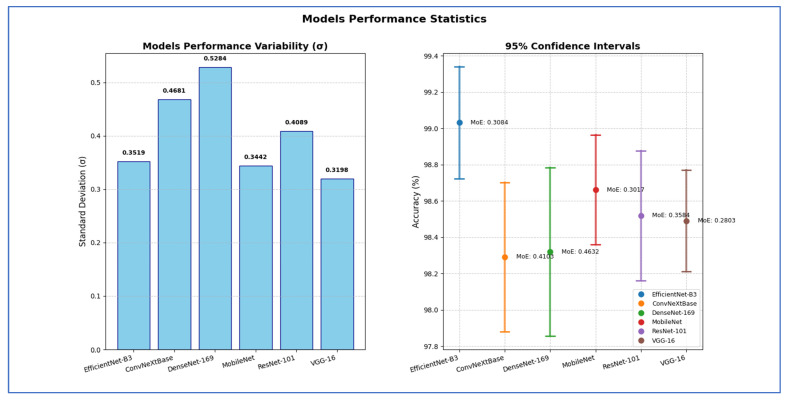
The standard deviation and CIs for the six DL models based on the accuracy through the five-fold cross-validation.

**Figure 19:**
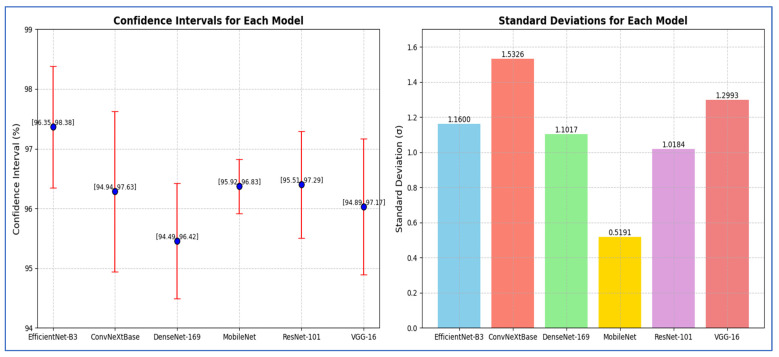
The standard deviation and CIs for the six DL models based on the F1-score through the five-fold cross-validation.

To evaluate the dependability of the results from EfficientNet-B3, a comprehensive statistical analysis was conducted. This analysis focused on the variance, standard deviation, standard error of the mean (SEM), and the confidence interval (CI) of the accuracy. The variance measures the average squared deviation of each data point from the mean of the data set. It quantifies how spread out the values are. The standard deviation is the square root of the variance. It expresses the average distance of the data points from the mean in the same units as the original data, making it more interpretable than variance. The SEM measures how much the sample mean differs from the actual population mean. It indicates the extent to which the sample mean would change if we collected multiple samples [28]. A CI is a range of values, derived from sample data, that is likely to contain the true population parameter (e.g., mean, accuracy) with a specified level of confidence (commonly 95% or 99%). CIs provide statistical reliability and robustness to model evaluation metrics (e.g., accuracy, F1-score, AUC). Instead of reporting a single number, CIs give a range that reflects uncertainty due to sample variation, which is particularly important in DL models trained on limited or imbalanced data [29]. The recorded measurements were obtained after performing the five-fold cross-validation of the procedure. The detailed evaluations of the six DL models for the EBTC dataset can be found in Table 10, Table 11 and Table 12, as well as Figure 17, Figure 18 and Figure 19.

Table 11 shows that the EfficientNet-B3 model delivered peak performance with a 99.03% mean accuracy (range: 98.58–99.72%), supported by moderate consistency (SD = 0.3519) and precise estimation (SEM = 0.1574) reflecting tight CIs.

The ConvNeXtBase achieved slightly lower mean accuracy (98.29%, range: 97.44–98.86%) with greater result dispersion, evidenced by higher variability metrics (SD = 0.4681, variance = 0.2192) and the poorest mean precision (SEM = 0.2094).

The DenseNet-169 attained 98.32% mean accuracy (range: 97.29–98.72%) but showed the most substantial performance fluctuations across trials, indicated by peak variability measures (SD = 0.5284, variance = 0.2792) and reduced mean reliability (SEM = 0.2363).

The MobileNet achieved robust 98.66% mean accuracy (range: 98.01–99.00%) with high result stability (SD = 0.3442) and confident mean estimation (SEM = 0.1540), demonstrating reliable performance.

The ResNet-101 yielded 98.52% mean accuracy (range: 98.01–99.15%) with moderate outcome spread (SD = 0.4089) and acceptable mean uncertainty (SEM = 0.1829).

The VGG-16 produced the most statistically stable results: 98.49% mean accuracy within the narrowest range (97.86–98.72%), minimal variability (lowest SD = 0.3198, variance = 0.1023), and the tightest mean confidence (lowest SEM = 0.1430).

Hence, EfficientNet-B3 delivered the highest average accuracy, while VGG-16 exhibited the most stable and consistent performance based on its low σ and SEM. DenseNet-169 and ConvNeXtBase showed the highest variability, implying less reliability across different test scenarios.

Table 12 shows that EfficientNet-B3 showed a standard deviation of 0.3519, indicating relatively moderate variation in performance across test runs. Its SEM was 0.1574, leading to a margin of error of 0.3084 and a confidence interval ranging from 98.72% to 99.34%. This implied the model achieved high accuracy with relatively tight precision.

The ConvNeXtBase exhibited a higher standard deviation (0.4681) and SEM (0.2094) than other models, resulting in a wider margin of error of 0.4103. The corresponding CI [97.88%, 98.70%] indicated greater uncertainty in its estimated performance, suggesting variability in results across different test sets.

The DenseNet-169 had the highest standard deviation (0.5284) and SEM (0.2363), which produced the widest margin of error of 0.4632 among all models. Its CI, ranging from 97.86% to 98.78%, showed that although its average performance was competitive, it suffered from significant variability, which reduced the reliability of its mean estimate.

The MobileNet maintained low variability, with a standard deviation of 0.3442 and a SEM of 0.1540, producing a margin of error of 0.3017. Its CI [98.36%, 98.96%] was among the narrower ones, indicating high precision and stability in its results.

The ResNet-101 had a standard deviation of 0.4089 and an SEM of 0.1829, yielding a margin of error of 0.3584. The confidence interval [98.16%, 98.88%] suggested moderate variability, and while the accuracy was fairly high, its reliability was slightly lower than that of MobileNet or VGG-16.

The VGG-16 showed the lowest variability overall, with the smallest standard deviation (0.3198), SEM (0.1430), and margin of error of 0.2803. The CI of [98.21%, 98.77%] was the narrowest, indicating that VGG-16’s performance was the most consistent and statistically stable among all models tested.

Hence, the EfficientNet-B3 attained the highest estimated accuracy with robust statistical precision. The VGG-16 exhibited the most dependable and consistent performance, showing the least uncertainty regarding its average accuracy. In contrast, the DenseNet-169 and ConvNeXtBase displayed more performance variability, indicating that their results may be more affected by different data splits or conditions.

From Table 13, the reported CI analysis based on F1-score demonstrated the reliability of performance metrics across the evaluated models. EfficientNet-B3 achieved the narrow CI range of [96.35, 98.38] with a margin of error of 1.02, indicating both high accuracy and stability. ConvNeXtBase showed a wider CI of [94.94, 97.63] and a larger margin of error (1.34), reflecting higher variability in its performance. DenseNet-169 produced a CI of [94.49, 96.42] with a margin of error of 0.97, suggesting moderate consistency. MobileNet exhibited the smallest margin of error (0.05) and the tightest CI [95.92, 96.83], indicating that it yielded the most stable performance among the models. ResNet-101 achieved a CI of [95.51, 97.29] with a margin of error of 0.89, reflecting balanced accuracy and reliability. VGG-16, on the other hand, produced a CI of [94.89, 97.17] with a margin of error of 1.14, suggesting greater variability compared to MobileNet and ResNet-101.

Overall, EfficientNet-B3 and MobileNet were characterized by the most consistent and robust results, while ConvNeXtBase and VGG-16 showed relatively higher variability.

Moreover, we implemented the paired t-test as shown in Table 14. From Table 14, the performance of different models was compared against the baseline model, EfficientNet-B3, which achieved an accuracy of 99.03%. All other models showed lower accuracy values. ConvNeXtBase reached 98.29%, representing a difference of −0.74%, and the difference was statistically significant. DenseNet-169 obtained an accuracy of 98.32%, with a difference of −0.71%, which was also statistically significant. MobileNet achieved 98.66%, differing by −0.37% from the baseline, and this difference was significant. ResNet-101 showed an accuracy of 98.52%, with a −0.51% difference, which was significant. VGG-16 achieved 98.49%, differing by −0.54% from EfficientNet-B3, and the difference was statistically significant. From this, the t-test gave t-statistic is 8.42 and *p*-value is 0.0011. The t-statistic of 8.42 indicated a substantial difference, suggesting that the performance of EfficientNet-B3 is consistently better than the other models. The *p*-value of 0.0011 < 0.05, implies that EfficientNet-B3’s accuracy is statistically significant higher than that of the other models.

Overall, EfficientNet-B3 consistently outperformed all other models, and the observed differences in accuracy were statistically significant at α = 0.05. This result confirms that EfficientNet-B3’s superior performance is not due to random chance and represents a meaningful improvement over the other architectures.

### 4.5. Component-Wise Performance Analysis

An ablation study is a structured experimental method used in DL to assess the impact of individual components or changes within a model architecture. This is done by selectively removing or modifying (ablating) parts of the model and observing how these changes affect performance. This approach helps to identify which elements—such as specific layers, data augmentation techniques, or hyperparameters—are crucial for the model’s effectiveness [30].

In the area of BLCA detection, ablation studies are especially valuable. They enable researchers to examine the effects of different design choices on diagnostic accuracy, sensitivity, and specificity. For instance, by testing variations of a convolutional network with and without attention mechanisms, it can be determined whether these modules significantly enhance tumor localization or classification performance [30].

In clinical AI applications, where dependability and clarity are vital, ablation studies offer insights that help optimize model architectures for real-world use while reducing issues like overfitting or redundancy. Ablation analysis is crucial for validating the effectiveness of designs in medical image classification models, particularly those used in cancer diagnosis tasks [30].

**Figure 20:**
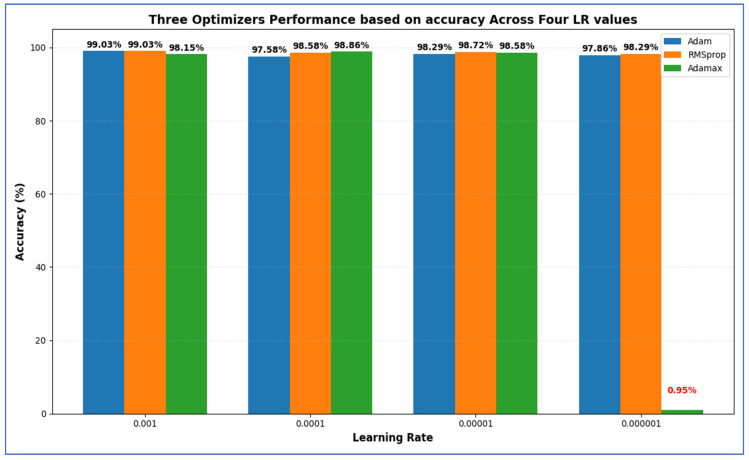
Three optimizers’ performance based on accuracy across four LR values, where the red value indicates the lowest accuracy.

In our experiment, we performed an ablation analysis on the test set of the EBTC dataset by adjusting the optimizers and learning rate (LR) configurations. We initially utilized the Adam optimizer with an LR set at 0.0001, achieving an accuracy of 99.03%. Next, we explored two more optimizers: Root Mean Square Propagation (RMSprop) and Adamax. For each optimizer, we evaluated LR values of 0.001, 0.0001, 0.00001, and 0.000001. The results indicating the performance metrics evaluation of EfficientNet-B3, obtained by ablating the above optimizers and LR values, are shown in Table 15 and illustrated in Figure 20.

Table 15 presents that among all configurations, the highest accuracy (99.03%) was attained by both Adam at LR = 0.001 and RMSprop at LR = 0.001, indicating that these settings were optimal for overall classification performance. RMSprop (LR = 0.001) also yielded the highest specificity (99.36%), showcasing its superior ability to correctly identify negative (non-cancer) cases, along with the lowest FNR (2.53%). This is particularly crucial in medical diagnoses to prevent missed cancer cases. In contrast, Adam (LR = 0.001) had a slightly higher FNR (3.15%) but achieved a higher precision (97.95%), indicating fewer FPs among predicted positives. The best recall (97.47%) was also recorded for RMSprop at LR = 0.001, suggesting that this model was most effective at accurately identifying true positive cases. Similarly, the highest F1-score (97.37%) was noted for the Adam optimizer with LR = 0.001, demonstrating a balanced efficiency between precision and recall.

In summary:

- Highest Accuracy: 99.03% (Adam & RMSprop, LR = 0.001).
- Highest Specificity: 99.36% (RMSprop, LR = 0.001).
- Lowest FNR: 2.53% (RMSprop, LR = 0.001).
- Highest NPV: 99.36% (Adam, LR = 0.001).
- Highest Precision: 97.95% (Adam, LR = 0.001).
- Highest Recall: 97.47% (RMSprop, LR = 0.001).
- Highest F1-Score: 97.37% (Adam, LR = 0.001).

Overall, both Adam and RMSprop at an LR of 0.001 performed the best, with RMSprop providing slightly better recall and specificity, while Adam achieved the highest precision and F1-score.

### 4.6. Discussion of Results in Light of Recent Advances

BLCA is recognized as a significant type of urological cancer, resulting in approximately 196,500 deaths. It ranks as the 9th leading cause of cancer deaths among men and the 19th among women [5,6]. The manual classification of muscular tissues by pathologists is a labor-intensive task that heavily relies on their expertise. This dependency can introduce variability among observers, particularly due to the similarities in the morphology of cancerous cells. Traditional methods for analyzing endoscopic images are often time-consuming and resource-intensive, complicating the efficient differentiation of various tissue types. Despite progress in early detection, robotic surgical techniques, and immunotherapy that have contributed to improved survival rates, BLCA continues to be a significant and increasing health concern globally, particularly in developed countries [1]. Consequently, there is a pressing need for a fully automated and reliable system to categorize BLCA images.

To tackle these challenges, this research presented a refined EfficientNet-B3 model specifically for detecting BLCA. This model aims to assist clinicians in identifying BLCA at an earlier stage, thereby decreasing both diagnostic time and costs. Additionally, the research employed a five-fold cross-validation method to improve the accuracy of the EfficientNet-B3 model. This method allows for effective parameter adjustments by partitioning the data into five subsets and repeating the process five times, with each subset serving as the validation set once. Five-fold cross-validation is a notable example of k-fold cross-validation, where (k) can be any integer greater than 1, with common values being 3, 5, or 10. This technique is widely recognized for providing a more accurate and reliable evaluation of DL models on unseen data.

The main goal of our experiment was to identify BLCA to enhance patient outcomes, streamline the diagnostic process, and significantly lower both patient time and costs. In our experiment, we utilized the EBTC dataset, which included a total of 1403 images. We allocated 80% of this dataset (1123 images) for training and reserved the remaining 20% (351 images) for testing. We implemented a supervised pre-training to pre-train six DL models—EfficientNet-B3, ConvNeXtBase, DenseNet-169, MobileNet, ResNet-101, and VGG-16—where the six DL models were trained on the ImageNet dataset.

Furthermore, we incorporated a five-fold cross-validation technique. This process involved dividing the training dataset into five equal subsets. In each iteration, one subset served as the validation set while the other four were used for training the model. Each iteration represented a distinct training and validation cycle that updated the model’s parameters. This procedure was repeated five times, with each subset acting as the validation set once. We calculated the average performance across all iterations to evaluate the model’s generalization capability.

Via the five-fold cross-validation, the EfficientNet-B3 achieved an accuracy between 98.58% and 99.72% (mean 99.03%) and a specificity from 98.97% to 99.80% (mean 99.31%), indicating exceptional discrimination of healthy cases. Its FNR varied from 0.45% to 4.43% (mean 3.15%), while the NPR value ranged from 99.04% to 99.79% (mean 99.36%), showing that negative predictions were almost always correct. Positive predictive value spanned 96.98% to 99.52% (mean 97.95%), and sensitivity ranged from 95.57% to 99.55% (mean 96.85%), with the F1-score balancing these at 96.50–99.53% (mean 97.37%). Overall, the model demonstrated outstanding specificity and precision, a low FNR, and robust accuracy, with only minor variability in recall across folds.

Maintaining an FNR between 0.45% and 4.43%, with an average of 3.15%, demonstrates high sensitivity. This means that the model rarely overlooks TP cases, enabling timely identification of patients with health conditions. As a result, earlier interventions can be made, leading to improved health outcomes. In healthcare, a low FNR minimizes the risk of denying necessary treatments to patients, which in turn lowers morbidity and mortality rates associated with delayed diagnoses. Additionally, a low FNR increases confidence in negative results, reducing the need for costly or invasive retests and improving the efficiency of patient management. From an algorithmic perspective, consistently maintaining a low FNR ensures that the model effectively detects positive cases across various datasets.

To evaluate the dependability of the results from EfficientNet-B3, a comprehensive statistical analysis was conducted. This analysis focused on the variance, standard deviation, and the CIs of the accuracy.

It is crucial to understand that the proposed system serves as a support tool rather than a substitute for human expertise. Every negative case flagged by the system must undergo clinical assessment, especially for high-risk individuals or ambiguous situations. Establishing thresholds informed by patient history, symptoms, and other risk elements may lead to additional testing or referrals to specialists for borderline cases. Moreover, consistently updating the model with new data from FN cases can reduce these mistakes over time.

**Figure 21:**
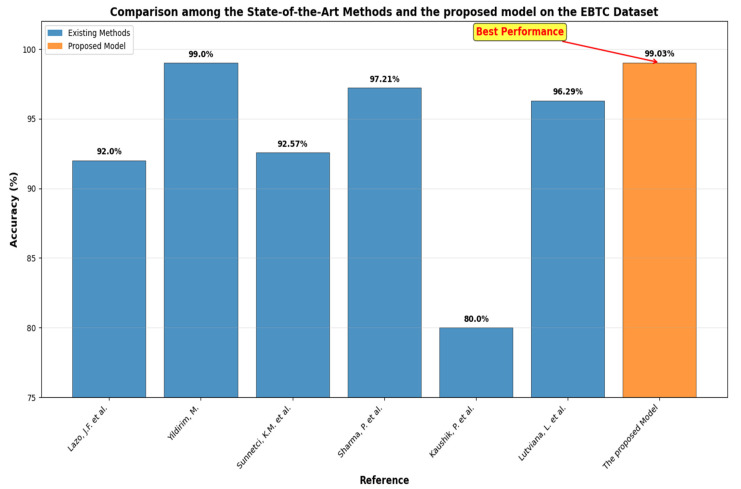
Performance comparison among the state-of-the-art methods and the proposed model [15,16,17,18,19,20].

Table 16 and Figure 21 show that the state-of-the-art studies consistently demonstrated strong classification performance on the EBTC dataset, but our EfficientNet-B3 approach outperformed them all. In Table 16, the references are limited to six because the dataset proposed in 2023 is the primary focus. Lazo et al. [15] attained 90% accuracy using a semi-supervised GAN, while Yıldırım [16] achieved 99.0% with a CBIR framework. Sunnetci et al. [17] evaluated various CNN-based and hybrid DL plus ML pipelines as well as a ViT, reaching 92.57%. Sharma et al. [18] combined a CNN with a Vision Transformer to yield 97.21%, and Kaushik et al. [19] reported 80% accuracy with a conventional CNN. Lutviana et al. [20] achieved 96.29% using another CNN architecture. In contrast, our model—fine-tuned EfficientNet-B3 with five-fold cross-validation—delivered 99.03% accuracy, surpassing prior methods in both mean performance and consistency across folds.

Compared to the approach in Yildirim, M. [16], which involved extracting deep features from multiple pre-trained CNNs—with DenseNet201 identified as the best feature extractor—and combining these 1000-dimensional vectors with traditional classifiers like Subspace KNN, as well as a content-based image retrieval CBIR pipeline, our method employed an end-to-end fine-tuned EfficientNet-B3 model. This model was trained directly on the raw images using five-fold cross-validation.

While Yildirim, M. [16] assessed seven CNN backbones along with texture descriptors (such as LBP and HOG) and seven similarity metrics to support both classification and CBIR tasks, our work was concentrated solely on enhancing classification performance with a single architecture. This focus allows us to achieve a comparable overall accuracy of 99.03% without the added complexity of feature-selection stages or external similarity-based retrieval. Furthermore, Yildirim, M. [16] processed images in their original form and separated feature extraction from classification, whereas our pipeline integrated image representation learning and decision-making into one cohesive model, thereby simplifying deployment and reducing preprocessing steps.

Our fine-tuned EfficientNet-B3 model outperforms the multi-stage pipeline in Yildirim, M. [16] for the following reasons:

1. EfficientNet employs compound scaling that balances depth, width, and resolution, resulting in significantly higher accuracy per parameter and FLOPS compared to traditional ConvNets. Specifically, EfficientNet-B3 outperforms ResNeXt-101 while using 18× fewer FLOPS, which leads to faster inference and reduced computing costs in deployment environments.
2. EfficientNet models are smaller yet more accurate, making them less susceptible to overfitting with limited datasets. Research indicates that EfficientNets achieve state-of-the-art accuracy on transfer-learning tasks with fewer parameters compared to larger architectures. This characteristic makes them particularly suitable when labeled data is scarce.
3. Instead of using pre-extracted deep features combined with classical classifiers as done in Yildirim, M. [16], our approach fine-tuned the entire EfficientNet-B3 backbone directly on BLCA images. This method enabled the network to learn domain-specific representations across all layers, often resulting in better classification performance compared to “frozen” feature plus separate classifier pipelines.

Optimizing our model’s performance has been a priority; however, achieving 100% accuracy remains difficult. This is due to several factors, including variability in imaging quality, differences in image scanners, and inherent limitations within the dataset. Additional challenges arise from noise, artifacts, and interobserver variability. Despite these obstacles, our model demonstrates competitive performance when compared to existing methods, and we have thoroughly assessed its accuracy using standard metrics. The proposed DL model has shown potential to outperform other recent classifiers, particularly following parameter tuning. We believe our approach lays a strong foundation for further advancements in this field.

### 4.7. Limitations and Future Work

Despite the promising results demonstrated by the proposed EfficientNet-B3-based framework, several limitations were observed. First, the model was trained and validated exclusively on the EBTC dataset, which, while valuable, comprised only 1403 images. This limited dataset size may constrain the model’s generalizability to broader clinical scenarios involving more diverse patient populations and imaging conditions. Additionally, the use of preprocessed data (resizing and normalization) may not fully capture real-world variations such as noise, artifacts, and lighting inconsistencies often present in clinical endoscopy.

Future work should aim to address these limitations by incorporating domain adaptation techniques or transfer learning from larger medical datasets, which may further bolster performance. The integration of explainable AI (XAI) methods, such as Grad-CAM or SHAP, could improve model transparency and clinician interpretability. Furthermore, real-time deployment and validation in clinical workflows should be pursued to assess feasibility, usability, and impact on diagnostic efficiency in real-world settings.

## 5. Conclusions

This study addressed the critical need for a reliable, automated system for classifying BLCA tissues from endoscopic images by proposing a refined EfficientNet-B3-based DL framework. The model was specifically developed to assist clinicians in accurately and efficiently identifying BLCA, thereby reducing diagnostic time, lowering costs, and improving patient outcomes. Leveraging the EBTC dataset containing 1403 images, pre-processed using techniques like resizing and normalization to ensure consistent input, the research employed supervised pre-training on six DL models and utilized a five-fold cross-validation approach to rigorously evaluate model performance.

The five-fold cross-validation strategy enabled effective parameter optimization and robust evaluation, contributing to the model’s strong generalization across unseen data. Furthermore, the ablation study and the statistical analysis—incorporating variance, standard deviation, and confidence intervals—validated the consistency and reliability of the results.

EfficientNet-B3 demonstrated superior results across multiple evaluation metrics. It achieved a mean accuracy of 99.03%, with specificity averaging 99.31%, indicating a strong capability in correctly identifying non-cancerous tissue. The model maintained a low FNR of 3.15%, highlighting its ability to correctly identify the majority of TP cases. This was crucial in minimizing missed cancer diagnoses and ensuring timely clinical intervention. Precision and recall averaged 97.95% and 96.85%, respectively, while the F1-score reached a mean of 97.37%, reflecting a well-balanced performance in handling both positive and negative predictions.

Overall, the proposed EfficientNet-B3 model outperformed conventional and hybrid architectures reported in the recent literature, offering a scalable and efficient solution for BLCA classification. Its ability to maintain high sensitivity and precision, along with a low FNR, underscores its potential for integration into real-world diagnostic workflows, particularly in settings where speed, accuracy, and resource efficiency are critical.

## Figures and Tables

**Table 1:** EBTC’s class distribution.

Class	Images Count
HGC	469
LGC	647
NTL	134
NST	504
Total	1754

**Table 2:** A comparative analysis between the EfficientNet-B3 and the other models.

Feature	EfficientNet-B3	VGG-16	ConvNeXtBase	MobileNet-V2	ResNet-101	DenseNet-169
Input Resolution	224 × 224	224 × 224.	224 × 224.	224 × 224.	224 × 224.	224 × 224.
Preprocessing	Standard normalization	Standard normalization	Standard normalization	Standard normalization	Standard normalization	Standard normalization
Core Building Block	MBConv + SE block (compound scaling)	Stacked conv layers + max pooling	ConvNeXt block (Conv + LayerNorm + GELU)	Inverted residual blocks	Residual blocks	Dense blocks (dense connections)
Low-Level Feature Extraction	Initial Conv + Pooling	Stacked Conv + ReLU + MaxPooling	Conv stem + large kernel conv	Conv + BatchNorm	Conv + BatchNorm	Initial Conv + Pooling
Mid-Level Feature Extraction	MBConv (depthwise + pointwise conv)	Sequential conv layers	Depthwise conv + pointwise conv (modernized ResNet-style)	Depthwise-separable conv	Residual bottleneck blocks	Dense blocks
High-Level Feature Extraction	Deeper MBConv layers	Deeper stacked conv layers	Larger receptive field ConvNeXt blocks	Bottleneck inverted residual blocks	Deep residual stacks	Deeper dense blocks
Regularization	Dropout + SE	Dropout + fully connected regularization	LayerNorm + DropPath (stochastic depth)	Dropout	Dropout	Dropout (optional)
Feature Fusion Strategy	Compound scaling (width, depth, resolution)	Sequential convolution and pooling	Hierarchical block scaling	Sequential bottlenecks	Residual skip connections	Dense feature concatenation
Classifier Head	Global Avg Pool + Dense	Flatten → Fully connected layers + Softmax	Global Avg Pool + Dense	Global Avg Pool + Dense	Global Avg Pool + Dense	Global Avg Pool + Dense
Domain Specialization	General-purpose	General-purpose (image classification benchmark)	General-purpose, modern ResNet replacement	Mobile/edge optimized	General-purpose	General-purpose

**Table 3:** The experiment’s hyperparameter.

Parameter	Value
img_size	224 × 224
Number of epochs	30
channels	3
Optimizer	Adam
Initial learning rate	0.001
Patience	10
loss	categorical_crossentropy

**Table 4:** The average of the cross-validation for EfficientNet-B3 when evaluated on the test set.

**EfficientNet-B3**	**Folds**	**Accuracy (%)**	**Specificity (%)**	**FNR (%)**	**NPV (%)**	**Precision (%)**	**Recall (%)**	**F1-Score (%)**
	1	98.58	98.97	3.93	99.04	96.98	96.07	96.50
	2	99.29	99.51	2.75	99.55	98.01	97.25	97.62
	3	98.86	99.20	4.19	99.26	97.28	95.81	96.50
	4	99.72	99.80	0.45	99.79	99.52	99.55	99.53
	5	98.72	99.06	4.43	99.16	97.98	95.57	96.67
	Average	99.03	99.31	3.15	99.36	97.95	96.85	97.37

**Table 5:** The average of the cross-validation for ConvNeXtBase when evaluated on the test set.

**ConvNeXtBase**	**Folds**	**Accuracy (%)**	**Specificity (%)**	**FNR (%)**	**NPV (%)**	**Precision (%)**	**Recall (%)**	**F1-Score (%)**
	1	98.29	98.78	4.13	98.83	96.67	95.87	96.23
	2	98.86	99.15	1.99	99.20	98.27	98.01	98.12
	3	98.43	98.82	3.37	98.93	97.68	96.63	97.12
	4	97.44	98.20	6.84	98.27	93.92	93.16	93.52
	5	98.43	98.90	3.35	98.93	96.26	96.65	96.44
	Average	98.29	98.77	3.94	98.83	96.56	96.06	96.29

**Table 6:** The average of the cross-validation for DenseNet-169 when evaluated on the test set.

**DenseNet-169**	**Folds**	**Accuracy (%)**	**Specificity (%)**	**FNR (%)**	**NPV (%)**	**Precision (%)**	**Recall (%)**	**F1-Score (%)**
	1	98.43	98.97	4.04	98.91	95.17	95.96	95.54
	2	98.72	99.19	2.07	99.07	95.32	97.93	96.48
	3	97.29	98.13	5.99	98.10	92.86	94.01	93.39
	4	98.72	99.09	4.37	99.14	97.09	95.63	96.32
	5	98.43	98.92	4.77	98.93	95.83	95.23	95.52
	Average	98.32	98.86	4.25	98.83	95.25	95.75	95.45

**Table 7:** The average of the cross-validation for MobileNet when evaluated on the test set.

**MobileNet**	**Folds**	**Accuracy (%)**	**Specificity (%)**	**FNR (%)**	**NPV (%)**	**Precision (%)**	**Recall (%)**	**F1-Score (%)**
	1	99.00	99.30	3.19	99.34	97.60	96.81	97.19
	2	98.72	99.13	2.96	99.12	95.90	97.04	96.43
	3	98.01	98.60	3.26	98.56	94.90	96.74	95.73
	4	98.72	99.11	4.52	99.18	96.41	95.48	95.92
	5	98.86	99.24	3.34	99.22	96.55	96.66	96.61
	Average	98.66	99.08	3.45	99.08	96.27	96.55	96.37

**Table 8:** The average of the cross-validation for ResNet-101 when evaluated on the test set.

**ResNet-101**	**Folds**	**Accuracy (%)**	**Specificity (%)**	**FNR (%)**	**NPV (%)**	**Precision (%)**	**Recall (%)**	**F1-Score (%)**
	1	98.01	98.58	6.08	98.66	95.97	93.92	94.86
	2	98.15	98.74	3.12	98.68	94.77	96.88	95.70
	3	98.72	99.09	3.61	99.16	96.69	96.39	96.51
	4	99.15	99.45	1.52	99.41	96.85	98.48	97.59
	5	98.58	98.99	2.30	98.96	96.99	97.70	97.34
	Average	98.52	98.97	3.32	98.98	96.25	96.68	96.40

**Table 9:** The average of the cross-validation for VGG-16 when evaluated on the test set.

**VGG-16**	**Folds**	**Accuracy (%)**	**Specificity (%)**	**FNR (%)**	**NPV (%)**	**Precision (%)**	**Recall (%)**	**F1-Score (%)**
	1	97.86	98.46	8.09	98.61	96.57	91.91	93.81
	2	98.72	99.03	2.97	99.13	98.15	97.03	97.55
	3	98.58	99.08	3.85	99.02	94.85	96.15	95.47
	4	98.72	99.14	2.94	99.12	95.87	97.06	96.43
	5	98.58	98.93	3.97	99.06	97.91	96.03	96.89
	**Average**	**98.49**	**98.93**	**4.36**	**98.99**	**96.67**	**95.64**	**96.03**

**Table 10:** A comparative performance for the six DL techniques.

Model	Peak Training Accuracy	Peak Validation Accuracy	Lowest Training Loss	Lowest Validation Loss	Performance Summary
EfficientNet-B3	~98%	~96%	~0.05	~0.10	Best performance overall; rapid convergence, high accuracy, and stable generalization.
ConvNeXtBase	~98%	~95%	~0.05	~0.15	Excellent results; high validation accuracy with slightly more initial fluctuations.
DenseNet-169	~98%	~94%	~0.05	~0.18	Very good performance; smooth convergence and robust generalization.
MobileNet	~97%	~93%	~0.07	~0.20	Strong accuracy and low loss, but slightly less stable validation performance.
ResNet-101	~98%	~94%	~0.06	~0.25	Good results; consistent accuracy but slightly higher fluctuations in validation loss.
VGG-16	~95%	~88%	~0.10	~0.50	Weakest performer; lower accuracy, higher loss, and significant instability during validation.

**Table 11:** The statistical analysis of the six DL models based on the accuracy using the test set of EBTC dataset.

Models	Avg (%)	Max (%)	Min (%)	σ	$\sigma^{2}$	SEM
EfficientNet-B3	99.03	99.72	98.58	0.351895	0.12383	0.157372
ConvNeXtBase	98.29	98.86	97.44	0.468139	0.219154	0.209358
DenseNet-169	98.32	98.72	97.29	0.528411	0.279219	0.236313
MobileNet	98.66	99.00	98.01	0.344246	0.118506	0.153952
ResNet-101	98.52	99.15	98.01	0.408909	0.167206	0.18287
VGG-16	98.49	98.72	97.86	0.3198	0.102272	0.143019

**Table 12:** The CIs of the six DL models based on the accuracy using the test set of the EBTC dataset.

Models	σ	SEM	CI	Margin of Error
EfficientNet-B3	0.351895	0.157372	[98.72289, 99.33979]	0.3084
ConvNeXtBase	0.468139	0.209358	[97.88026, 98.70094]	0.4103
DenseNet-169	0.528411	0.236313	[97.85592, 98.78226]	0.4632
MobileNet	0.344246	0.153952	[98.35922,98.96271]	0.3017
ResNet-101	0.408909	0.18287	[98.16009, 98.87694]	0.3584
VGG-16	0.3198	0.143019	[98.20971, 98.77035]	0.2803

**Table 13:** The CIs of the six DL models based on the F1-score using the test set of EBTC dataset.

Models	σ	SEM	CI	Margin of Error
EfficientNet-B3	1.159983194	0.518760255	[96.34895, 98.38249]	1.016770099
ConvNeXtBase	1.5326	0.685399	[94.9438, 97.630593]	1.343382916
DenseNet-169	1.101654602	0.492675	[94.4859, 96.41722]	0.965643
MobileNet	0.519134	0.232164	[95.917704, 96.827]	0.045504
ResNet-101	1.018366	0.455427	[95.50543, 97.2907]	0.892637
VGG-16	1.299307	0.581068	[94.89062, 97.1684]	1.138893

**Table 14:** The pairwise approximate *p*-value matrix.

Models	Accuracy	Difference	*p*-Value	Statistically Significant (α = 0.05)
EfficientNet-B3	99.03	Baseline	**-**	
ConvNeXtBase	98.29	0.74	0.0011	Yes
DenseNet-169	98.32	0.71	0.0011	Yes
MobileNet	98.66	0.37	0.0011	Yes
ResNet-101	98.52	0.51	0.0011	Yes
VGG-16	98.49	0.54	0.0011	Yes

**Table 15:** The EfficientNet-B3’s performance metrics evaluation by ablating three optimizers and four LR values on the test set of the EBTC dataset.

Model	LR	Accuracy (%)	Specificity (%)	FNR (%)	NPV (%)	Precision (%)	Recall (%)	F1-Score (%)
Adam	0.001	99.03	99.31	3.15	99.36	97.95	96.85	97.37
	0.0001	97.58	98.30	11.19	98.58	96.07	88.81	91.11
	0.00001	98.29	98.76	7.02	98.95	97.24	92.98	94.70
	0.000001	97.86	98.52	7.15	98.61	94.99	92.85	93.80
RMSprop	0.001	99.03	99.36	2.53	99.34	96.71	97.47	97.06
	0.0001	98.58	98.99	4.31	99.08	96.53	95.69	96.09
	0.00001	98.72	99.09	3.94	99.15	97.28	96.06	96.63
	0.000001	98.29	98.81	6.53	98.94	95.76	93.47	94.50
Adamax	0.001	98.15	98.83	4.39	98.74	93.61	95.61	94.47
	0.0001	98.86	99.19	4.33	99.29	97.49	95.67	96.51
	0.00001	98.58	99.02	5.41	99.11	96.27	94.59	95.37
	0.000001	0.95	0.95	0.95	0.95	0.95	0.95	0.95

**Table 16:** Performance comparison with state-of-the-art methods.

Reference	Methodology	Accuracy	Datasets
Lazo, J.F. et al. [15]	semisupervised GAN	92%	EBTC dataset
Yildirim, M. [16]	CBIR	99.0%	EBTC dataset
Sunnetci, K.M. et al. [17]	CNNs, CNN-ML or DL + ML, and ViT	92.57%	EBTC dataset
Sharma, P. et al. [18]	combined CNN with a ViT	97.21%	EBTC dataset
Kaushik, P. et al. [19]	CNN	80%	EBTC dataset
Lutviana, L. et al. [20]	CNN	96.29%	EBTC dataset
The proposed Model	EfficientNet-B3 and five-fold cross-validation	99.03%	EBTC dataset

## Data Availability

The original data presented in the study are openly available in https://www.kaggle.com/datasets/aryashah2k/endoscopic-bladder-tissue-classification-dataset (accessed on 16 March 2023).
